# Effects of acidification on the proteome during early development of *Babylonia areolata*

**DOI:** 10.1002/2211-5463.12695

**Published:** 2019-07-31

**Authors:** Guilan Di, Yanfei Li, Guorong Zhu, Xiaoyu Guo, Hui Li, Miaoqin Huang, Minghui Shen, Caihuan Ke

**Affiliations:** ^1^ College of Fisheries Henan Normal University Xinxiang China; ^2^ State Key Laboratory of Marine Environmental Science College of Ocean and Earth Sciences Xiamen University Xiamen China

**Keywords:** *Babylonia areolata*, larva, ocean acidification, pCO_2_, proteomics

## Abstract

Increases in atmospheric CO_2_ partial pressure have lowered seawater pH in marine ecosystems, a process called ocean acidification (OA). The effects of OA during the critical stages of larval development may have disastrous consequences for some marine species, including *Babylonia areolata* (Link 1807), a commercially important sea snail in China and South East Asia. To investigate how OA affects the proteome of *Babylonia areolata*, here we used label‐free proteomics to study protein changes in response to acidified (pH 7.6) or ambient seawater (pH 8.1) during three larvae developmental stages of *B. areolata*, namely, the veliger larvae before attachment (E1), veliger larvae after attachment (E2), and carnivorous juvenile snail (E3). In total, we identified 720 proteins. This result suggested that acidification seriously affects late veliger stage after attachment (E2). Further examination of the roles of differentially expressed proteins, which include glutaredoxin, heat‐shock protein 70, thioredoxin, catalase, cytochrome‐c‐oxidase, peroxiredoxin 6, troponin T, CaM kinase II alpha, proteasome subunit N3 and cathepsin L, will be important for understanding the molecular mechanisms underlying pH reduction.

**Abbreviations**
ACN: acetonitrile
AGC: automatic gain control
ANOVA: analysis of variance
DTT: dithiothreitol
E1: Veliger larvae before attachment
E2: Veliger larvae after attachment
E3: Carnivorous juvenile snail
FDR: false discovery rate
GO: gene ontology
HCD: high‐energy collision dissociation
MS: mass spectrometry
OA: ocean acidification
PCA: principal component analysis
pCO_2_: CO_2_ partial pressure
PP: polypropylene
STEM: short time‐series expression miner
TCA: trichloroacetic acid
TFA: trifluoroacetic acid

The gradually increasing atmospheric CO_2_ partial pressure (pCO_2_) has lowered the seawater pH in marine ecosystems. This change in seawater pH is regarded as ocean acidification (OA). The pH of the ocean's surface waters is predicted to decrease by 0.3–0.4 units by the year 2100 1. An estimated reduction of up to pH 7.3 in sea water is expected within the next 300 years 1, 2, 3.

Marine shellfish is a key community that stabilizes the marine ecosystem, is considered as the driving force of the ocean material flow and energy flow, and plays an important role in the marine carbon cycle 4. The monetary value of OA impact on overall shellfish production is estimated to be about 100 billion USD 5. The effects of a changing living environment on larvae are important, especially because these early development stages often represent a bottleneck for species struggling to survive in the changing marine environment 6, 7. The effects of OA during the critical stages of larval development may have disastrous consequences for some marine species 8, for instance, inhibition of the fertilization, embryonic and larval development, and survival in the acidified seawater of Sakura clam (*Macoma balthica*) 9. Gazeau *et al*. 10 found that OA reduces the hatching and adhesion rates of mussels (*M. edulis*). OA can delay early embryo and larval development and show teratogenic and lethal effects in many marine invertebrates 11, including California mussels (*Mytilus californianus*) and *Ruditapes decussatus* 12, 13. The reductions of 0.7 units often lead to an abnormal development and high mortality in some shellfish, for instance, *C. gigas* 11, *Mytilus galloprovincialis* 14, 15, and *Pinctada martensii* 16.

Differences in growth and larval development time may have important consequences for population dynamics and demography 17; larval developmental plasticity is a crucial source of variation in adult phenotypes and can directly influence the evolution of populations and species 18, 19. Larval development includes a pelagic larval phase and benthic phase in some gastropod; larvae undergo metamorphosis and transited to benthonic larval phase and juveniles 20. The attachment and metamorphosis of gastropod larvae affect the quantity change, population distribution, and species evolution 21. Therefore, investigation on changes of gastropod larval development in pH may provide a realistic indication of how shellfish respond to OA.

Overall, OA can negatively affect the main physiological processes of shellfish, OA can influence calcification, destroy the acid–base balance of organisms, change metabolism, and reduce the immune function. However, the current related research methods are mainly based on traditional physiology and ecology 22, but lack studies which focus on the molecular response mechanism. Using molecular ‘‐omics’ techniques to reveal the molecular signals underlying the response of development characteristic to changes in pH is now possible 23. Proteomics can be a comprehensive and accurate method to analyze the response mechanism of marine organisms to OA, the fitness of an organism can be assessed by proteome‐molecular traits, which are directly related to their physiological performance 24. Therefore, identification and characterization of proteins involved in various metabolic processes are required to predict the capacity of the organism to adapt to OA.

In the past decade, the environmental proteomics studies lead to the identification of molecular pathways and differentially expressed proteins of shellfish in response to OA 25, 26. Proteomic studies have shown that elevated *p*CO_2_ (resulting in pH 7.3–7.87) leads to the differentially abundant proteins associated with oxidative stress, protein synthesis, energy metabolism, and the cytoskeleton in the oysters *Crassostrea virginica*,* C. hongkongensis*,* C. gigas,* and *Saccostrea glomerata* 24, 25, 26, 27, 28, 29, 30, 31, 32, 33, and affects energy and primary metabolisms, stress responses, calcium homeostasis, the nucleotide metabolism, and cytoskeleton structure in pacific oyster *Crassostrea gigas* 34. Identifying these proteins as well as determining the effect that OA exerts on oysters is fundamental to understand the mechanisms involved 25. Dineshram *et al*. 29 examined the effects of OA on the proteome of the oyster species *Crassostrea hongkongensis*; over 7% of the proteome was altered in response to OA at pH 7.6. Proteomic analysis of wild‐type *C. gigas* and B2 *S. glomerata* (a selective breeding line) showed that most of identified proteins were upregulated in the wild‐type population. However, these proteins were downregulated in the B2 *S. glomerata* by CO_2_ stress (pH 7.5–7.84) 26. OA could reduce the expression level of some proteins to conserve energy, but OA could induce oxidative stress proteins 35. Some of these OA‐responsive molecules and pathways have been identified in marine invertebrate organisms, including oysters 24, 25, 36.

In proteomic of pCO_2_ on larvae development, proteomics was used to compare the global protein expression pattern of oyster larvae exposed to ambient and to high CO_2_ 27. Dineshram *et al*. 28 proteomic studies revealed a set of proteins, referred to as ‘protein expression signature’, that changed their expression when the larvae were exposed to OA. Comparative proteome analysis data confirmed that there was a significant suppression of molecular pathways involved in protein degradation, energy production, and tissue growth, which correlate with decreased larval metamorphic behavior after the pH stress at 7.4, and more than 11% proteins of larval were downregulated with decreased pH 30. Similarly, a study about the proteome in an earlier larval stage of *C. gigas* showed that a third of expressed proteins were downregulated in response to decrease pH 27. Larval forms of tubeworms, barnacles, and oysters also showed increased downregulation of proteins associated with calcification, energy production, and cytoskeleton in response to decreased pH (pH 7.6) 37, 38. However, the specific function of these proteins in larva as a tolerance molecular mechanism to decreased pH is unknown.

The ivory shell, *B. areolata,* is the most promising economic marine gastropod in the present century due to their wide distribution along most parts of Asian coastlines and the southeast coast of mainland China, their delicate flavor, and high market reception. The annual output of *B. areolata* is more than 1000 tons, and the worth is more than 100 million RMB Yuan 39, 40.

In this study, three different stages of larvae of *B. areolata* were cultured at high (OA) and ambient CO_2_ (control) conditions, larvae of similar physiological age and size from the OA and the control groups were analyzed by label‐free quantitative proteomics, and differentially expressed proteins were identified by mass spectrometry (MS). We investigated OA on the larvae of *B. areolata* as a model to address the following questions. How does the larval proteome respond to OA? Which proteins are involved in the response? Our objectives were as follows: This label‐free quantitative proteomics approach will enable us to partially elucidate as to how calcification, metabolism, oxidative stress, and stress tolerance proteins were modified in response to OA. Furthermore, to our knowledge, there is no other study to date using proteomics to differentiate OA‐exposed *B. areolata* larval proteome versus healthy controls. Proteomics can identify crucial proteins involved in larval developmental phenotypes in response to future changes in pH and provides insights into the potential mechanisms involved in stress response to and tolerance of high CO_2_ levels.

## Materials and methods

### Experimental apparatus and organisms

The construction of seawater CO_2_ system was advocated as previously described 41, which was the study result of our laboratory. The pCO_2_ manipulation system is shown in Fig S1. The methods are briefly described as follows: The principle of experimental system was to mix CO_2_‐free, dry air, and pure CO_2_ (99.99% purity) together at different ratios using mass flow rates to produce CO_2_‐enriched air with different pCO_2_ 42. Atmospheric air was provided from an oil‐free, medical air compressor. The water and particles were removed through two filters (GFR600‐25, AIRTAC), then two polypropylene (PP) columns filled with soda lime to absorb the CO_2_ were connected, and then a similar PP column filled with anhydrous CaCl_2_ to further remove water was connected again. Finally, a 5‐cm disk‐type air filter was connected before connecting the regulating utilities. The treated air was then delivered into a pressure regulation valve and then into a needle valve, which can maintain a stable air flow.

Mass flow of CO_2_ and air was measured by two mass flow sensors and was set to the desired level of CO_2_ results in a CO_2_ concentration of 800 ppm by adjusting the needle valves. The pCO_2_ manipulation and measurement system were performed as previously described in the literature (see our previous study 41). The method is described as follows (see Fig S1): both CO_2_ and air were homogenized at the bottom of a plastic container to generate CO_2_‐enriched air with different CO_2_ concentrations. A small proportion of these gas mixtures were directly monitored with a CO_2_ detector (Li‐7000, LI‐COR) through a bypass. Further adjustment of the needle valve was carried out before the reading system of the detector achieved the preset standard. The mass flow sensors and CO_2_ detector were connected to a computer, and measurements were recorded with the associated software. The majority of CO_2_‐enriched air was mixed with filtered seawater (0.22 μm filtered). Two small holes were drilled in the lid of the plastic container for input and outlet of CO_2_‐enriched air. The container was kept semiclosed in order to ensure that the inner space of the container was filled with CO_2_‐enriched air and the used seawater which was separated from the ambient air. Carbonate system parameters were calculated using co_2_sys software (version 2.1, Oak Ridge, TN, USA). Temperature and pH were measured with a pH meter. A total of 2 CO_2_ concentrations were used: 400 ppm, which represents the current CO_2_ concentration, and 2000 ppm 43, which represents the predicted CO_2_ concentrations for the year 2300.

### Samples acquisition

Adult *B. areolata* broodstock were purchased from the Haicang farm, Fujian province, and individuals with vitality were selected. Snails were maintained in an ecological culture pond (27.3 ± 1.1 °C, salinity 30.1 ± 0.9, pH 8.0 ± 0.65, oxygenated water). The snails were fed daily with oysters and chopped fresh fish. All experiments involving animals reported in this study were approved by the Animal Care and Use Ethics Committee of the Xiamen University.

After *B. areolata* broodstock spawning, the oocysts were washed with filtered seawater and then transferred to 40‐mL sample bottles containing filtered seawater with different pH values (pH 7.6 and 8.1). One oocyst was placed in each sample bottle and changed sea water one time each day until the larvae hatched from the oocysts. In order to ensure the stable pH value of the water, the sample bottle was put into the preservation box and CO_2_‐enriched air was input into the box. The incubation time was recorded, and the samples were collected after the larvae were hatched from the oocyst. Under continuous exposure, embryos were cultured at different CO_2_ concentrations from fertilization until the end of the experiment. For *B. areolata*, fertilization, hatching, and developmental conditions were measured.

In this study, three developmental stages of *B. areolata*, the veliger stage of *B. areolata* before attachment, the late veliger stage of *B. areolata* after attachment, and the juvenile stage (see our previous study 40) were sampled. When larva is the veliger larval prior to settlement stage, many changes ensue, including the loss of velum and gill development. Then, with the development of foot, the larvae crawled and began their feeding. When morphological changes occurred, with the transition stage from planktonic larvae stage to benthic stage, that was from the late veliger stage to the juvenile stage, the feeding habits of *B. areolata* also gradually changed from phytophagy to sarcophagy.

The larvae of every developmental stages were divided into five replicates. The control group, C, was at 400 ppm, and pH was at 8.15 ± 0.01, whereas the experimental group, E, was at 2000 ppm and pH at 7.61 ± 0.02. The water temperature was kept at 27 °C. For the veliger stage before attachment, the control and acidified group were named C1 and E1, respectively; for the late veliger stage of metamorphosis at the bottom (velum atrophy), the control and acidified group were named C2 and E2, respectively; for the carnivorous juvenile snail, the control and acidified group were named C3 and E3, respectively; three larvae stages are shown in Fig S2A–C. Early veliger larvae were fed daily with algae. When the late veliger stage sinks to the substratum and begins a benthic life, the feeding habits of larvae also gradually changed from phytophagy to sarcophagy; then, the snails were fed daily with oysters. Under these culture conditions, >85% of embryos developed into early veliger stage in 60 h, after which, the seawater was replaced with new seawater, larvae were cultured for 2 days, and then the veliger larva enters the late veliger larva stage. After 11–12 d, the late veliger stage enters the juvenile snail stage. Larval samples with similar physiological age and shell size between the control and the OA treatment were used for comparative proteome analysis. For every development stage, five replicates were set at each pCO_2_ level. Three biological duplicates (triplicates) were selected from each of the five replicates for proteome analysis. Samples collected three development stages fulfilled with those similar larval size and age requirement for proteome analysis.

In every development stage, almost all individuals were in the same development period by microscopic examination. Approximately 4000–5000 of larvae per stage, fresh and living larvae, were filtered using an 80‐μm filter, and then, the larvae were collected into 1.5‐mL tubes for further analysis. The samples were centrifuged at 13 000 ***g*** for 3 min at 4 °C. The larval material (15 μL of pellets) was stored at −80 °C. For every development stage, the larvae were divided into three independent samples, and three replicates were performed for each development stage to ensure reproducibility.

### Total protein extraction with TCA/acetone

A total of 100 mg sample was suspended immediately in 1 mL 10% TCA/acetone (*w*/*v*) solution, mixed, and then stably placed over 2 h at −20 °C. The sample was centrifuged at 17 000 ***g*** for 15 min at 4 °C; the supernatant was removed and then added with 1 mL 10% TCA/acetone (*w*/*v*) solution with 20 mm DTT. The sample was solubilized with a sonicator (BRANSON, S‐450D), the sample was placed on ice, the program of sonicator was run by using 5–10 min blasts of 15% amplitude lasting 5 s each, with 4 s breaks, and then stably placed at 4 °C for 30 min. Samples were fully homogenized with a tissue homogenizer and stably placed at 4 °C for 30 min. The sample was centrifuged at 17 000 ***g*** for 15 min at 4 °C, and the supernatant was removed. Then, the protein pellet was washed three times using 80% acetone solution with 20 mm DTT and added 1 mL each time. The sample was placed at 4 °C for 30 min. Then, the sample was centrifuged at 17 000 ***g*** for 15 min at 4 °C; the upper layer was removed, and the lower layer pellet was sampled. The precipitation block was carefully agitated with sterile toothpick every time. The protein pellet was washed with 1 mL 20 mm DTT 100% acetone and then placed at 4 °C for 30 min. The sample was centrifuged at 17 000 ***g*** for 15 min at 4 °C; then, the supernatant was removed, and the protein pellet was sampled. Then, the protein pellet concentration was redissolved in solution (6 m urea, 2 m thiourea, 0.1% SDS, 1 mm DTT, 10 mm Hepes, pH 8.0). The concentration of protein was measured using a Protein 2‐D Quant kit (GE Healthcare, Piscataway, NJ, USA).

### Digestion of proteins

In brief, 50 μg of proteins was alkylated with 55 mm iodoacetamide for 30 min at room temperature in the dark. Then, proteins were incubated with trypsin (1 g/50 g protein) (Promega, Madison, WI, USA) for 12 h at room temperature. The digestion of proteins was stopped by adding 5% formic acid (the final concentration was about 0.1%).

### Desalting with C18 tips

Desalination was done using prewet tips with 100 μL of 50% acetonitrile (ACN) solution; the tips were discarded after. This step was repeated one time. The tips were then balanced with 100 μL of 0.1% trifluoroacetic acid (TFA) and repeated once. The digestion sample of 100 μL was sucked using the tips and then blown out; this step was repeated more than 10 times. Finally, the samples were blown out, and then, 5% acetonitrile containing 0.1% TFA was sucked with the tips, repeated once and followed elution in 100 μL of 50% acetonitrile containing 0.1% formic acid. The collected eluate was washed two times every time; 100 μL 95% acetonitrile containing 0.1% formic acid was sucked. After concentration and drying of elution liquid, the sample was then fully dissolved with 30 μL liquid (2% methanol and 0.1% formic acid) by vortex; polypeptide concentration was measured with Nanodrop microspectrophotometer, stored at −70 °C, or centrifuged at 14 000 ***g*** for 15 min, and then directly analyzed.

### Strong cation exchange liquid chromatography

The first dimension was separated using SCX column; the flow rate was 1000 μL·min^−1^, divided into 10 compositions according to the chromatographic peak, then desalted by C18 Cartridge and lyophilization, and then frozen at −80 °C. Gradient of liquid chromatography is set as follows: 0–25 min, 100% buffer A (KH_2_PO_4_ 10 mmol·L^−1^ pH 3, 25% acetonitrile); 25–35 min, 90% buffer A, 10% buffer B (KH_2_PO_4_ 10 mmol·L^−1^ pH 3.0, 500 mmol·L^−1^ KCl, and 25% acetonitrile); 35–45 min, 80% buffer A, 20% buffer solution B; 45–50 min, 55% buffer A, 45% buffer B; 50–60 min, 100% buffer B; and 60–75 min, 100% A buffer.

### Nanoacquity ultraperformance liquid chromatography

The two‐dimensional sample was separated using Easy nLC liquid‐phase system. The sample was dissolved with 50 μL solution (0.1% formic acid, 5% acetonitrile) and injected with autosampler into the Zorbax 300SB‐C18 peptide traps (Agilent Technologies, Wilmington, DE, USA). Then, the sample was separated using chromatographic column; the column flow rate is set to 200 nL·min^−1^. Solution A (0.1% formic acid aqueous solution) and solution B (0.1% formic acid acetonitrile water solution, in which acetonitrile was 100%) were used as the mobile phases with the gradient elution procedures. The liquid‐phase gradient was as follows: 0–100 min, the range of B linear gradient was 5%–30%; 100–130 min, the range of B linear gradient was 30%–40%; 130–135 min, the range of B linear gradient was 40%–90%; and 135–140 min, the range of B was maintained at 90%. Chromatographic column was 75 μm × 150 mm (RP‐C^18^), which was equilibrated with 95% solution A.

### Q‐Exactive mass spectrometric protein identification

Raw MS/MS data files were processed to peak lists in proteome discoverer software (Thermo Fisher Scientific, software version 1.4, Waltham, MA, USA) and then submitted to Mascot‐generated files searches (Matrix Science, London, UK). The data‐dependent tandem mass spectrometry label‐free analysis was carried out on a Q‐Exactive mass spectrometer (Thermo Fisher Scientific, MA, USA); the flow rate was 300 nL·min^−1^. Full scan spectra were acquired with a maximum ionization time of 200 ms, the spectra were recorded at 70 000 resolution, and the AGC target value was set as 10^6^ ions; the number of scan ranges was 1, and then, 10 most abundant peaks for MS/MS were selected using a 18 000 resolution scan (the AGC target value was set as 1 × 10^4^ ions; the maximum ionization time was 200 ms) with a 1.5 m/z ion selection window, and a normalized collision energy of 30% was used for collision‐induced dissociation. A 40 s dynamic exclusion window was used to avoid repeated selection of peptides for MS/MS. MS2 activation type: HCD; MicroScan: 1; Underfill ratio: 0.1%.

### Analysis of the Q‐Exactive mass spectrometric data

Thermo Fisher Scientific Orbitrap RAW files were converted into complete peak lists, RAW spectra were converted into Mascot‐generated files (mgf) using proteome discoverer software (Thermo Fisher Scientific, software version 1.4) and analyzed by using sieve software (Thermo Fisher Scientific)  to quantify all detected peaks. All data were acquired based on 99% confidence for protein identification by the false discovery rate (FDR) ≤ 1%. Statistical analysis was carried out using a one‐way ANOVA. *P*‐values ≤ 0.05 acquired by Tukey's test were considered significant. The *q*‐value was used to estimate false‐positive results. Peaks exhibited expression changes (>1.5‐fold). The search parameters were as follows: the enzyme specificity was trypsin; database: gastropods; the allowance was two missed cleavage site; the variable modification was Met oxidation; the fixed modification was carbamidomethyl (cysteine); protein mass was unrestricted; monoisotopic mass values were obtained; the fragment mass tolerance was ± 0.2 Da; and the peptide mass tolerance was ± 30 ppm. Protein FDR ≤ 0.05.

### Bioinformatics analysis

The Gene Ontology (GO) database provides a controlled vocabulary for describing genes and gene products, as well as provides full access to this information in several formats. UniProt Knowledgebase (UniProtKB) was used for protein function analysis and acts as the central hub of protein knowledge by providing a unified view of protein sequence and function. Currently, UniProtKB database included more than 78 037 000 terms. The web site is http://www.uniprot.org/uniprot/.

## Results

### Principal component analysis

**Figure 1:**
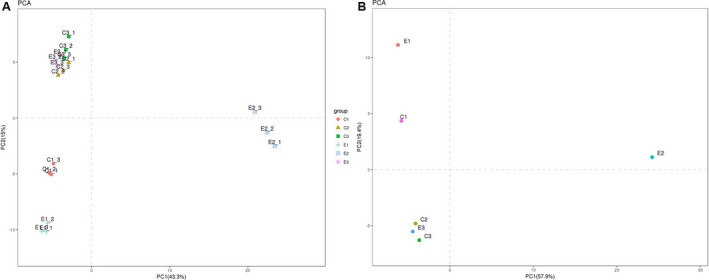
Principal component analysis (PCA) of six groups samples. (A) represents PCA including three biological replicate samples; (B) represents PCA analysis of mean in each group.

Principal component analysis (PCA) is a statistical procedure that converts a set of observations of possibly correlated variables. The differentially expressed proteins characteristic parameters in six groups were analyzed by PCA (Fig 1); principal component 1 (PC1) and principal component 2 (PC2) were obtained. The load value of six groups characteristics to two principal components and the variance contribution proportion of two principal components are shown in Fig 1A,B. In Fig 1A, the contribution proportion of PC1 was 43.3%, the contribution proportion of PC2 was 15.0%, and the PC1 and PC2 cumulative contribution proportion was 58.3%, biological duplicate samples were clustered together, the results showed that there was little difference between intragroups, and there was good repeatability between biological duplication samples. The results were extremely variable between C2 and E2 (Fig 1B), suggested that C2 larva stage was sensitivity to OA, and acidification seriously affects late veliger larvae after attachment.

### Identification of differentially expressed proteins

In this study, a total of 720 proteins (FDR < 1%, at least two unique peptides) were identified, and 245 differential abundance proteins (>1.5‐fold) were acquired. As shown in Table 1, the protein abundance of E group is regarded as upregulation or downregulation. The results showed that the total number of differentially expressed proteins was 148 between C2 and E2 groups, whereas the number of proteins was 57 between C1 and E1 and was 26 between C3 and E3. Differentially expressed proteins were determined using normalized % protein amount, with protein identifications summarized in Tables S1–S3.

**Table 1:** Numbers of differentially expressed proteins at different development stages after acidification

	C1/E1	C2/E2	C3/E3
Total differential protein	57	148	26
Downregulation	19	46	17
Upregulation	38	102	9

For C1 and E1, eight differentially expressed proteins that increased over five times in E1, such as histone 2A (12.5‐fold), glutaredoxin (7.14‐fold), tubulin beta chain (6.25‐fold), cathepsin D (5.56‐fold), huntingtin interacting protein K (5.56‐fold), catalase (5.56‐fold), and cytochrome‐c‐oxidase subunit I (5.26‐fold). Seven differentially expressed proteins that decreased over three times in E1 included tumor rejection antigen‐like protein (17.37‐fold), 26S protease regulatory subunit 6B (8.36‐fold), pedal peptide precursor protein (3.97‐fold), endo‐1,3‐beta‐D‐glucanase (3.2‐fold), tropomyosin (3.11‐fold), and troponin T (3.02‐fold) (Tables S1).

For C2 and E2, 15 differentially expressed proteins that increased over 34 times in E2 included pol‐like protein (1022.28‐fold), BiP/GRP78 (303.99‐fold), ATP synthase F0 subunit 6 (162.13‐fold), ribosomal protein L10a (154.53‐fold), KRP‐A (123.59‐fold), histidine decarboxylase (110.98‐fold), ADP‐ribosylation factor 2 (107.50‐fold), thioredoxin (85.70‐fold), Src tyrosine kinase 1 (35.97‐fold), and ferritin (34.64‐fold). Thirteen differentially expressed proteins that decreased over seven times in E2 included histone H4 (50.07‐fold), heat‐shock cognate protein 70 (23.13‐fold), H(+)‐transporting two‐sector ATPase alpha subunit (19.30‐fold), histone macro2A.1 (15.47‐fold), CaM kinase II alpha (11.59‐fold), arginine kinase (11.38‐fold), malate dehydrogenase precursor (10.61‐fold), tumor rejection antigen‐like protein (10.55‐fold), 60S ribosomal protein L18 (9.45‐fold), alpha tubulin 2 (7.08‐fold), and glutathione S‐transferase isoform (7.07‐fold) (Tables S2).

For C3 and E3, nine differentially expressed proteins that increased over 2.20 times in E3 included tumor rejection antigen‐like protein (28.75‐fold), RAB protein (2.99‐fold), protein disulfide isomerase (2.86‐fold), Adh3 (2.47‐fold), twitchin‐like protein (2.26‐fold), and manganese‐superoxide dismutase (2.20‐fold). Thirteen differentially expressed proteins that decreased over 2.40 times in E3 included hemocyanin (11.34‐fold), importin beta 1 (3.31‐fold), thyroid peroxidase‐like protein (2.87‐fold), psmc6 protein (2.61‐fold), mitochondrial malate dehydrogenase precursor (2.57‐fold), and ATP synthase, H+ transporting, mitochondrial F1 complex, and o subunit (2.40‐fold) (Table S3).

**Figure 2:**
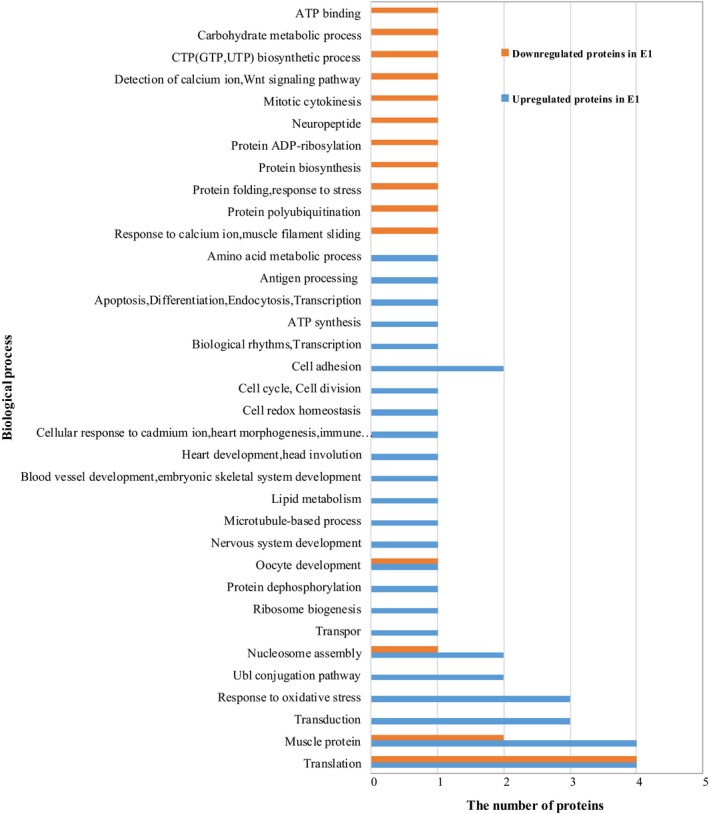
Gene ontology analysis for differentially abundant proteins identified by MS/MS with function and process classifications between C1 and E1.

For C1 and E1, identification of differentially expressed proteins involved in the biological process is summarized in Fig 2. The upregulated proteins in E1 were involved in response to oxidative stress, transduction and head involution. The downregulated proteins in E1 were involved in protein polyubiquitination, detection of calcium ion, carbohydrate metabolic process, and ATP binding.

For C2 and E2, identification of differentially expressed proteins involved in the biological process is summarized in Table S4. The upregulated proteins in E1 were involved in response to oxidative stress, head involution, protein glycosylation, protein dephosphorylation, lipid metabolism, and ATP synthesis; the downregulated proteins in E2 were involved in stress response and immunity.

**Figure 3:**
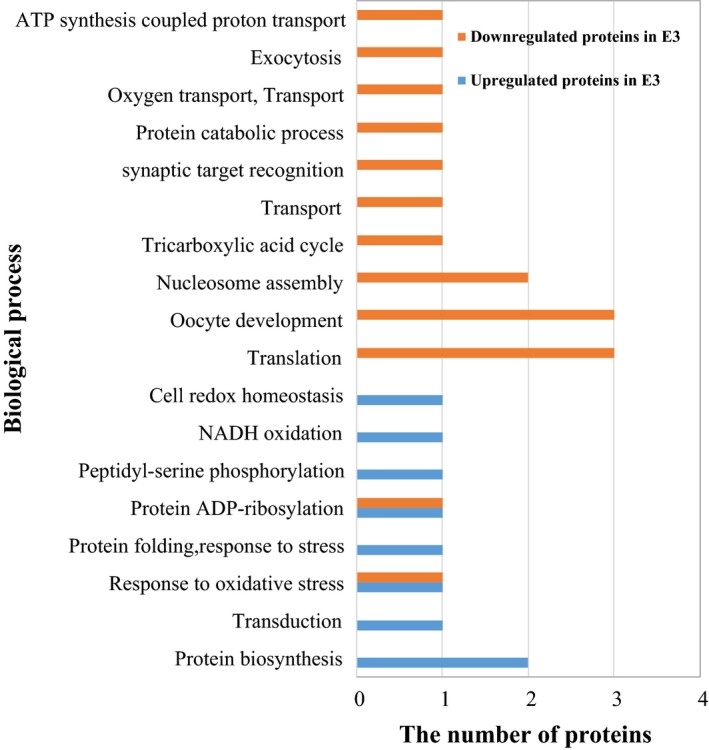
Gene ontology analysis for differentially abundant proteins identified by MS/MS with function and process classifications between C3 and E3.

For C3 and E3, identification of differentially expressed proteins involved in the biological process is summarized in Fig 3. The upregulated proteins in E3 were involved in protein biosynthesis, NADH oxidation, and transduction; the downregulated proteins in E3 were involved in signal translation and tricarboxylic acid cycle.

### Series protein expression data analysis by the Short Time‐series Expression Miner

Short time‐series expression miner is a tool for the analysis of short time‐series gene (or protein) expression data 44, which can also analyze protein expression. The data from the *B. areolata* larva sampled at six groups (C1, C2, C3, E1, E2, and E3) were filtered to contain the 245 differentially expressed proteins that exhibited 1.5‐fold upregulation or downregulation for at least one sample; the number of types was set 20. Comparing with the C1, all samples relative to C1 were calculated the log2 value.

**Figure 4:**
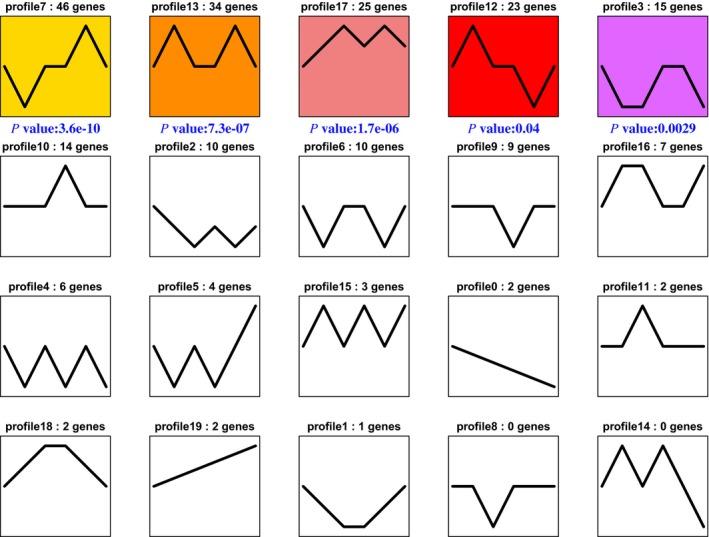
Reordered interface for model profiles by the Short Time‐series Expression Miner (STEM). Data were obtained from six groups (C1, C2, C3, E1, E2, and E3). Dataset contained 245 differential proteins. Clusters ordered based on the number of proteins and profiles arranged by significance. Colored profiles show statistically significant numbers; *P* value was arranged, ‘genes’ in the graph refer to ‘proteins’ data.

**Figure 5:**
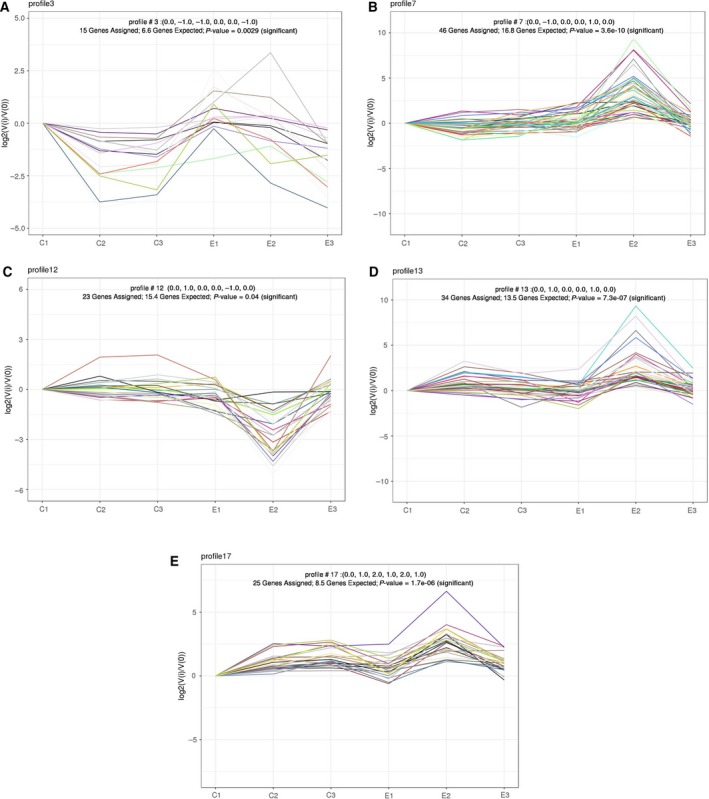
Detailed information on five model profiles by STEM. A‐E Images are five model profiles for 3, 7, 12, 13, and 17, respectively.

Along each window are statistics on the number of proteins which were assigned to the profile and the enrichment *p*‐value. The number at top of a profile box shows the profile ID number. The colored profiles had a statistically significant number of proteins assigned (‘genes’ in the graph refer to ‘proteins’ data). Colored profiles of the same color represent profiles grouped into a single cluster (Fig 4). The bottom of each colored window shows profiles with *P*‐value enrichment for proteins annotated (*P*‐value < 0.05). Differentially expressed proteins in profiles 3, 7, 12, 13 and profiles 17 were significant (Fig 5). Differentially expressed proteins in profiles 3, 7, 12, 13 and profiles 17 were summarized in Tables S5–S9.

For profile 3, proteins with differential expression included ATP synthase beta subunit, putative mitochondrial ATP synthase F chain, mitochondrial malate dehydrogenase precursor, gelsolin, endo‐1, 3‐beta‐D‐glucanase, p38 MAPK, actin, putative tubulin beta chain, tektin A1, axonemal dynein light‐chain p33, collagen pro alpha‐chain, munc18‐1‐interacting protein 1, poly‐(ADP‐ribose) polymerase I, and non‐neuronal intermediate filament protein A.

For profile 7, differentially expressed proteins included sodium/potassium ATPase alpha subunit, hemocyanin, proteasome subunit N3, cathepsin L‐like cysteine proteinase, histone H2A isoform 2, thioredoxin, alcohol dehydrogenase, ATP synthase F0 subunit 6, defender against apoptotic cell death 1, glucose‐regulated protein 78 kDa, ADP‐ribosylation factor 2, 17 beta‐hydroxysteroid dehydrogenase type 11, thioredoxin peroxidase 1, ubiquitin‐conjugating enzyme, es1 protein, ferritin, NCAM‐related cell adhesion molecule, histidine decarboxylase, Src tyrosine kinase 1, mitochondrial malate dehydrogenase precursor, thyroid peroxidase‐like protein, LIM protein, cadherin‐like 3, indoleamine dioxygenase‐like myoglobin, vitellogenin, protein disulfide isomerase, putative polyadenylate‐binding protein 1, putative RNA‐binding protein, ribosomal protein S4, 40S ribosomal protein S3a, ribosomal protein l5, 60S ribosomal protein L15, ribosomal protein l17, ribosomal protein L28, vasa‐like protein, actin, alpha tubulin 1, BiP/GRP78, cytoplasmic fragile X interacting protein, KRP‐A, pol‐like protein and myosin heavy chain type II.

For profile 12, differentially expressed proteins included heat‐shock cognate protein 70, PL10‐like protein, chaperonin containing T‐complex polypeptide subunit zeta, voltage‐dependent anion channel 2‐like protein, histone macro2A.1, histone H4, histone 1.1, arginine kinase, H(+)‐transporting two‐sector ATPase alpha subunit, malate dehydrogenase precursor, galectin, cytosolic malate dehydrogenase precursor, RAB2, CaM kinase II alpha, catalytic subunit of protein kinase A, myosin II heavy chain, myosin heavy chain, ribosomal protein S9, putative 60S ribosomal protein L3, ribosomal protein S6, 60S ribosomal protein L18, nucleoside diphosphate kinase B, and elongation factor 1 alpha.

For profile 13, differentially expressed proteins included calmodulin, troponin T, l‐3‐hydroxyacyl‐coenzyme a dehydrogenase, Cu/Zn‐superoxide dismutase, prohibitin‐2, Cdc24‐like protein, NaK‐ATPase alpha subunit, sodium/potassium ATPase alpha subunit, mitochondrial ATP synthase delta chain, cytochrome‐c‐oxidase subunit 1, signal sequence receptor beta‐like protein, glutathione S‐transferase, Bip‐like protein, mitochondrial malate dehydrogenase precursor, pedal peptide precursor protein, QM‐like protein, actin ovestestis isoform, beta tubulin, tropomyosin, dynein light chain, myosin heavy‐chain type II, elongation factor 1 alpha, guanine nucleotide‐binding protein G(q)‐alpha subunit, ribosomal protein S14, 60S ribosomal protein L31,40S ribosomal protein S16, ribosomal protein L10a, vitelline envelope zona pellucida domain 10 and vitelline envelope zona pellucida domain 7.

For profile 17, differentially expressed proteins included H2, histone H3, histone 2B, ATP synthase, ATP‐dependent RNA helicase DDX5, H+ transporting, mitochondrial F1 complex, o subunit, triosephosphate isomerase, psmc6 protein, calcineurin A, snail soma ferritin, huntingtin interacting protein K, ubiquitin‐like protein, cathepsin L, actin A1, myosin heavy‐chain type II, myosin essential light chain, guanine nucleotide regulatory protein beta subunit, splicing factor arginine/serine‐rich 4, ribosomal protein L26, 40S ribosomal protein S29, vitelline envelope zona pellucida domain 2 type 7 protein, vitelline envelope zona pellucida domain 8 and vitelline envelope zona pellucida domain protein 21.

### Hierarchical cluster analysis

**Figure 6:**
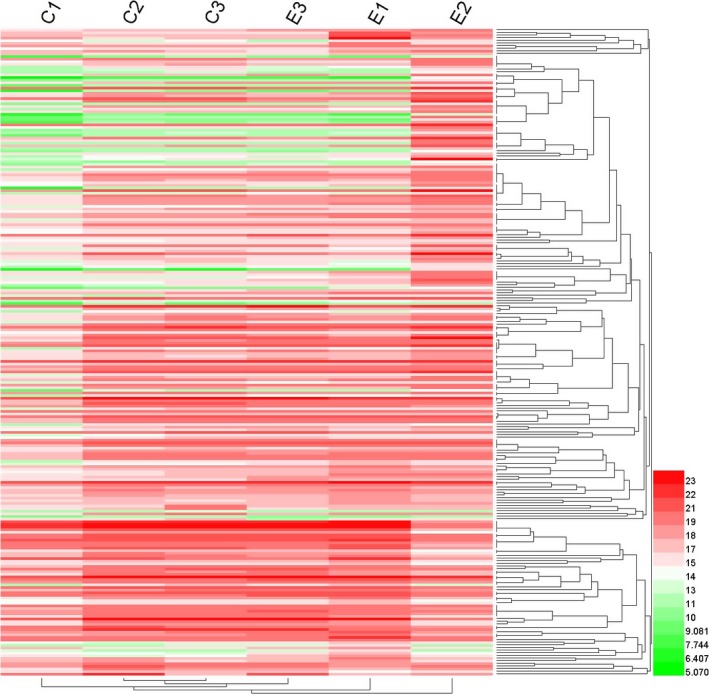
A Dendrogram of hierarchical cluster analysis on C1, C2, C3, E1, E2, and E3.

Hierarchical cluster analysis was used to examine the patterns of similarities and differences among genotypes. Protein abundant hierarchical clustering was performed to identify main classes of protein abundance. Distances were determined for the six groups (C1, C2, C3, E1, E2, and E3) with a TMEV heatmap, and a dendrogram was constructed using hemi 1.0 statistical software. Distances were calculated based on protein value intensities in all technical replicates. In this study, 720 proteins were detected; we selected a mean value‐based approach and then proceeded with analysis. Results showed that C2 and C3 clustered in one clade. The result showed that formed clusters indicated clear separation of E2 from other groups (Fig 6).

## Discussion

Label‐free quantitative proteomics provided rigorous and powerful tools for analyzing protein changes. In the present study, 720 proteins were identified. The low rate of solid attribution may have been caused by the limited cDNA information available for *B. areolata* or by improper annotations of *B. areolata* genes. *B. areolata* is a nonmodel species; the tryptic digestion resulted in acceptable MS/MS spectra; however, these mass signals did not match any peptide available 45 in the *B. areolata* or other gastropods identifiable protein databases. In this study, the number of differentially expressed proteins between C2 and E2 was largest; this finding suggests that acidification seriously affects late veliger larvae after attachment.

### Effects of OA on proteins associated with respiration and oxidative stress

In this study, the expression level of differential proteins associated with oxidative stress increased in E1 and E2; NADH is an important material for cell respiratory metabolism, and NADH oxidation function was upregulated in E3. The result showed that the number of upregulation proteins which are located on the mitochondria is larger than that of downregulation proteins in E1, E2, and E3.

The previous study showed that the metabolic process (including food intake, oxygen consumption, and ammonia excretion) of three bivalves: the pearl oyster *Pinctada fucata*, the noble scallop *Chlamys nobilis,* and the green‐lipped mussel *Perna viridis* was likely affected by the reduction in seawater pH 46. Fernández‐Reiriz *et al*. 47 found that the acidification conditions tested significantly reduced the respiration rates and increased the ammonia excretion rate of*Ruditapes decussatus* seeds. Zhang *et al*. 48 found that respiration rate of *Chlamys farreri* also decreased significantly at pH 7.3. Ding *et al*. 49 found that the respiratory metabolism pathway and the concerning enzymes of marine organisms might be changed as ocean acidification development. Those studies suggest that marine organisms have a series of adaptive measures to rescue the negative effects of low pH on respiration; these measures include increasing breathing frequency, expanding the depth of breathing, oxygen consumption increased first and then decreased, and a series of complex physiological regulation mechanism.

In the present study, cytochrome‐c‐oxidase subunit I increased 5.26‐fold in E1; cytochrome‐c‐oxidase is the terminal enzyme in the electron transport chain reaction. Thompson *et al*. 26 showed that proteins involved in the mitochondrial electron transport chain and oxidative stress were the most affected by OA. In the identification of proteins between E1 and C1, the amount of glutaredoxin increased 7.14‐fold among the upregulated proteins in E1, and catalase increased 5.56‐folds in E1. Between E2 and C2, thioredoxin increased 85.70‐fold in E2. Glutaredoxin and thioredoxin are considered to be a typical antioxidant, and thioredoxin can also remove the hydrogen peroxide; the catalase catalyzes the decomposition of hydrogen peroxide into oxygen and water.

Matoo *et al*. 50 studies have already demonstrated the negative impacts of elevated temperature and CO_2_ levels on the metabolism and cell oxidation in these bivalve species. The combined effects of decreased pH and increased temperature values on antioxidant enzyme activities and lipid peroxidation were evaluated in the clam *Chamelea gallina* and the mussel *Mytilus galloprovincialis* 51. Superoxide dismutase, catalase, and glutathione S‐transferase activities as well as lipid peroxidation were measured. The results demonstrated that the experimental conditions significantly influenced the biochemical parameters of the bivalves 51.

In this study, peroxiredoxin 6 increased 2.04‐fold in E1 (Table S1), peroxiredoxins (Prxs) work as a redox sensor to resist peroxide attacks. Previous studies showed that upregulation of some Prxs can protect cells from oxidative damage 52. A similar study also showed the significant variation of Prxs in mantle of eastern oyster *C. virginica* and hepatopancreas of pacific oyster *C. gigas* under elevated pCO_2_ condition using proteomic analysis 34, 35. Therefore, the upregulation of Prx 6 perhaps suggested the stress responses to elevated pCO_2_ exposure, especially oxidative stress.

### Effects of OA on proteins associated with response to immunity

Two differential expression proteins were identified as heat‐shock protein 70, which decreased 23.13‐fold and 5.50‐fold in E2, respectively (Table S2). Dineshram *et al*. 30 showed that decreased pH caused the reduction of several heat‐shock proteins (Hsps), and the downregulation of Hsps has also been documented in barnacles under decreased pH 37 and sea urchins 53.

In GO of the biological process involved the identification of proteins. In comparison with C2, the protein involved in the stress response and immune function in E2 decreased. Organisms in the early developmental stages, namely, larval and juvenile, have low immunity that may be affected by the degree of acidification 54. Gazeau *et al*. 10 and Matozz *et al*. 51, 55 showed that acidification can significantly reduce the immunity in *Mytilusedulis*,* Chamelea gallina,* and *M. galloprovincialis* and decrease the rate of metabolism. Michaelidis *et al*. 56 showed that water pH below 7.5 was harmful on the immune function of mollusk; this result leads to a permanent reduction of hemolymph pH value, growth, and metabolism; however, the level of hemolymph bicarbonate from shell dissolution increased. Berge *et al*. 57 showed that acidification made a negative effect on the immunity and growth of shellfish; they also suggested that the decline of immune function was related to the negative metabolism. Prolonged exposure to pH reduction of 0.5–0.7 pH units possibly elicits strong stress responses in many organisms 15.

We also identified a number of proteins involved in the metabolism of proteins, carbohydrates, and fats. These metabolisms are related to the acid–base balance in the organism. Acidification can change the acid–base balance in mollusks, and acid–base regulation is closely related to the internal immune defense function.

### Effects of OA on proteins associated with biological calcification and mineralization

In this study, for the differentially identified proteins involved in the calcification biological process, in comparison with E1 and C1, troponin T decreased 3.02‐folds in E1, and the abundance of troponin T protein was decreased in gills of pacific oyster *Crassostrea gigas* 34. CaM kinase II alpha decreased 11.59‐fold in E2. The biological process of detection of calcium ion was downregulated in E1. In the subcellular localization of identification protein, a portion of the upregulated proteins in E1 was found to be in the calcineurin and troponin complexes in E2; these complexes are closely related to the regulation of calcium.

In previous studies, OA can negatively affect calcification 58. Larvae of mollusks may be particularly at risk of acidification in marine environments 59 because larval shells are fragile 60. In OA, biomineralization of gastropods and bivalves is especially vulnerable in mollusk embryonic development and larval growth stage. Biocalcification requires the formation of early skeletal structures, which renders larvae sensitive to the reductions in the saturation state of carbonate ions 61

In calcifying shellfish species, the calcification rate was generally reduced in decreased pH 59. The mineral formation and calcification mechanism of mollusks and sea urchin larvae also indicate their sensitivity to OA 62. The decrease of 0.3 pH units resulted in slow growth and calcification of *C. gigas* during the first 3 days of larval development 63; the growth and metamorphosis of the *C. gigas* larvae were significantly reduced in decreased pH 64. Miller *et al*. 65 found that the larval shell area of *Crassostrea virginica* decreased 16% in the range of pH 7.7–8.1; the proportion of calcium in the shells decreased nearly half. OA made larvae shells of the *M. galloprovincialis* and *P. martensii*to grow slowly and become deformed 14, 48, 66. *Ruditapes philippinarum* had slightly dissolved calcium shell under the conditions of acute CO_2_ acidification 67.

In addition, Dineshram *et al*. 30 showed that the expression rates of over 24 proteins involved in calcification were changed under decreased pH by comparative proteome analysis, the majority of those proteins were downregulated, including calmodulin and several calcium binding proteins. In previous studies, proteins involved in calcification and acid–base regulation were downregulated under decreased pH in sea urchins 68, 69, bivalves 27, 28, and other marine invertebrates 70, 71, 72, 73. The results suggested that acidification can cause the response of calcium‐related proteins in the development of the *B. areolata* larva.

### Effects of OA on proteins associated with metabolism

In the present study, the downregulated proteins included proteins, which are involved in carbohydrate metabolic process and ATP binding process in E1. The downregulated proteins in E3 included proteins, which are involved in tricarboxylic acid cycle. In this study, differential proteins involved in energy metabolism included ATP synthase, malate dehydrogenase precursor, arginine kinase, and triosephosphate isomerase. The result showed that proteins related to the metabolism of the snail larvae have changed under the condition of acidification.

Lower invertebrate animals are easily affected by OA due to their restricted metabolism and physiology 54. Michaelidis *et al*. 56 found that the protein synthesis function and respiratory energy of the Mediterranean mussel (*M. galloprovincialis*) can be reduced if they live in sea water with low pH for a long time. In the pH range of 7.20–8.10, the metabolic substrates are mainly carbohydrates in the thick shell mussel, and no significant difference in the respiration entropy was found; this finding shows that no significant effect on the energy metabolism of the thick shell mussel in this pH range exists. When pH was 7.0, a significant change in energy metabolism was observed. Lannig *et al*. 74 found that the metabolic pathways of Pacific oysters (*C. gigas*) have changed under the influence of OA. As marine acidification intensifies, the metabolic pathways of *C. farreri* also changed 47. Some species have a strong ability to maintain the acid–base balance. In the face of OA, maintaining the stability of pH value requires a lot of energy; thus, species need to strengthen their metabolism to provide energy. Some species have a weak ability to maintain the acid–base balance. Given the insufficient ability to compensate the effects of pH change, before the appropriate environmental conditions recover, species have to reduce material and energy consumption by reducing the metabolic intensity to maintain their survival 75.

In this study, the proteins involved in protein glycosylation, protein dephosphorylation, lipid metabolism, and ATP synthesis were upregulated in E2. Dineshram *et al*. 27 found that vacuolar H [+]‐ATPase was seen upregulated that indicated that oyster larvae respond to OA by altering its cellular proton transportation at the expense of ATP. In the present study, when E2 was compared with C2, arginine kinase decreased 11.38‐fold in E2, arginine kinase catalyzes the reversible transfer from ATP to arginine to form phosphoarginine, there is a negative correlation between arginine kinase abundance and ATP concentration, implying the reduction of energy consumption 34. In this work, downregulation of malate dehydrogenase (MDH) in E2 (Table S2) perhaps illustrates the influence of elevated pCO_2_ exposure on energy metabolism 34. The proteins involved in protein biosynthesis were upregulated in E3. In this study, acidification pH was about 7.6; the substrate of energy metabolism in *B. areolata* probably had changed from carbohydrates into protein and lipids with increasing larval development and acidification time; the results need to be further studied and discussed. If the metabolic substrate is converted to protein, then this conversion can lead to break more muscle tissues down for metabolism; this result was detrimental to the healthy growth of mollusks. In the present study, the differentially expressed proteins also included actin, tubulin, and myosin; the result can be related to muscle metabolism.

Following exposure to CO_2_ stress, marine calcifiers often exhibit a decrease in metabolic activity in an attempt to conserve energy, which in turn leads to lower calcification rates and somatic growth 36, 54, 74, 76. Dineshram *et al*. 30 showed that larvae showed to undergo metabolic suppression in response to decreased pH, while larvae maintained their important cellular functions and metabolic pathways suppressed, including amino acid, carbohydrate metabolism, and deactivation of neuronal signaling. Ko *et al*. 64 have also shown similar result in larval growth and metamorphosis. These proteins changes reflect a changed energy allocation strategy that may make the *B. areolata* larvae to survive in response to decreased pH. This study has support from a recent larval proteomics study in the *C. gigas*) 30.

#### Other proteins

In this work, proteasome subunit N3 and cathepsin L in E2 (Table S2) were upregulated in E2; ubiquitin‐conjugating enzyme was upregulated in E1 and E2. Proteasome is mainly responsible for the degradation of abnormal proteins and many cellular regulatory proteins through ubiquitin pathways. Cathepsin L played a major role in protein degradation 77; in this study, cathepsin L was upregulated in E2 under elevated pCO_2_ exposure. The results perhaps indicated that the elevated pCO_2_ exposure increased the degradation processes of abnormal proteins.

## Conclusions

In the present study, we investigated the proteomic and phenotypic responses of *B. areolata* larvae in response to OA. This finding suggests that acidification seriously affects veliger larvae after attachment. The identification of differential abundance of key proteins, such as glutaredoxin, heat‐shock protein 70, thioredoxin, catalase, cytochrome‐c‐oxidase, peroxiredoxin 6, troponin T, CaM kinase II alpha, proteasome subunit N3, and cathepsin L during early development, is important to understand molecular mechanisms in the larval developmental of *B. areolata* in environments with reduced pH. However, further proteomic studies are required to validate these results. Moreover, information about protein abundance must be integrated with preceding patterns of gene expression, such as RT–PCR, and measures of physiological quality to better understand the mechanisms that can permit mollusk larvae to cope with future pH change. Proteome analysis and the novel proteins identified for future studies can extend our understanding of the physiological adaptation of shellfishes to OA.

## Acknowledgements

This work was supported by the Chinese Ministry of Science and Technology through the National Key Research and Development Program of China (2018YFD0901400). The Earmarked Fund for Modern Agro‐industry Technology Research System (No. CARS‐49), National Marine Economic Development Demonstration Project in Xiamen (No. 16CZB023SF12), Platform for Germplasm Sharing of Characteristic Aquaculture Species in Fujian, China scholarship council (CSC), the Projects of the Science and Technology Department of Henan Province (182102110328), the Key Project of Science and Technology Research of Henan Provincial Department of Education (No. 14B240003), Innovative Research Team (in Science and Technology) in University of Henan Province (15IRTSTHN018), Innovation Scientists and Technicians Troop Construction Projects of Henan Province CXTD2016043.

## Conflict of interest

The authors declare no conflict of interest.

## Author contributions

CH Ke and GL Di conceived and designed the study. GL Di, XY Guo, YF Li, and H Li performed the experiments, data analysis, prepared figures, and manuscript writing. GR Zhu analyzed the data. MQ Huang and MH Shen collected larval materials and edited the manuscript. All authors have read and approved the manuscript.

## Supporting information


**Fig. S1.** pCO_2_ manipulation system (Guo *et al*., 2015). pCO_2_ manipulation system with parts as follows 1: air compressor, 2: filter, 3: soda lime column, 4: CaCl_2_ anhydrate column, 5: disc‐type air filter, 6 and 10: pressure regulation valve, 7 and 11: needle valve, 8 and 12: mass flow sensor, 9: CO_2_ cylinder, 13: plastic jar, 14: CO_2_ detector, 15: computer. Solid line: air or CO_2_ flow; dashed line: digital signal transferring to computer.
**Fig. S2.** Embryonic development of *B. areolata*. (A) Veliger before attachment; (B) Veliger at the late metamorphosis stage (velum atrophy); (C) Juvenile *B. areolata*.

**Table S1.** Differentially expressed proteins with important physiological functions between C1 and E1.

**Table S2.** Differentially expressed proteins with important physiological functions between C2 and E2.

**Table S3.** Differentially expressed proteins with important physiological functions between C3 and E3.

**Table S4.** Differentially expressed proteins with function and process classifications between C2 and E2.

**Table S5.** Differentially expressed proteins enrichment analysis table for profile 3.
**Table S6.** Differentially expressed proteins enrichment analysis table for profile 7.
**Table S7.** Differentially expressed proteins enrichment analysis table for profile 12.
**Table S8.** Differentially expressed proteins enrichment analysis table for profile 13.
**Table S9.** Differentially expressed proteins enrichment analysis table for profile 17.
