# Supplementary material for: Effects of acidification on the proteome during early development of Babylonia areolata
Source: FEBS Open Bio. 2019 Jul 31;9(9):1503–20. doi: 10.1002/2211-5463.12695 (PMC6722889; doi:10.1002/2211-5463.12695)

**Titles and legends of figures**

**Supplementary Figures 1** pCO_2_ manipulation system (Guo et al., 2015)

pCO_2_ manipulation system with parts as follows 1: air compressor, 2: filter, 3: soda-lime column, 4: CaCl_2_ anhydrate column, 5: disc-type air filter, 6 and 10: pressure regulation valve, 7 and 11: needle valve, 8 and 12: mass flow sensor, 9: CO_2_ cylinder, 13: plastic jar, 14: CO_2_ detector, 15: computer. Solid line: air or CO_2_ flow; dashed line: digital signal transferring to computer

**Supplementary Figures 2** Embryonic development of *B. areolata*

(A) Veliger before attachment; (B) Veliger at the late metamorphosis stage (velum atrophy); (C) Juvenile *B. areolata*

**Supplementary Figure 1**


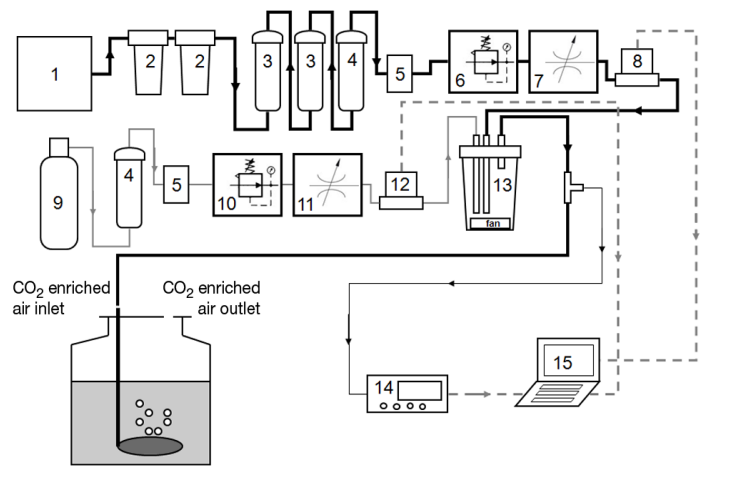


**Supplementary Figure 2**


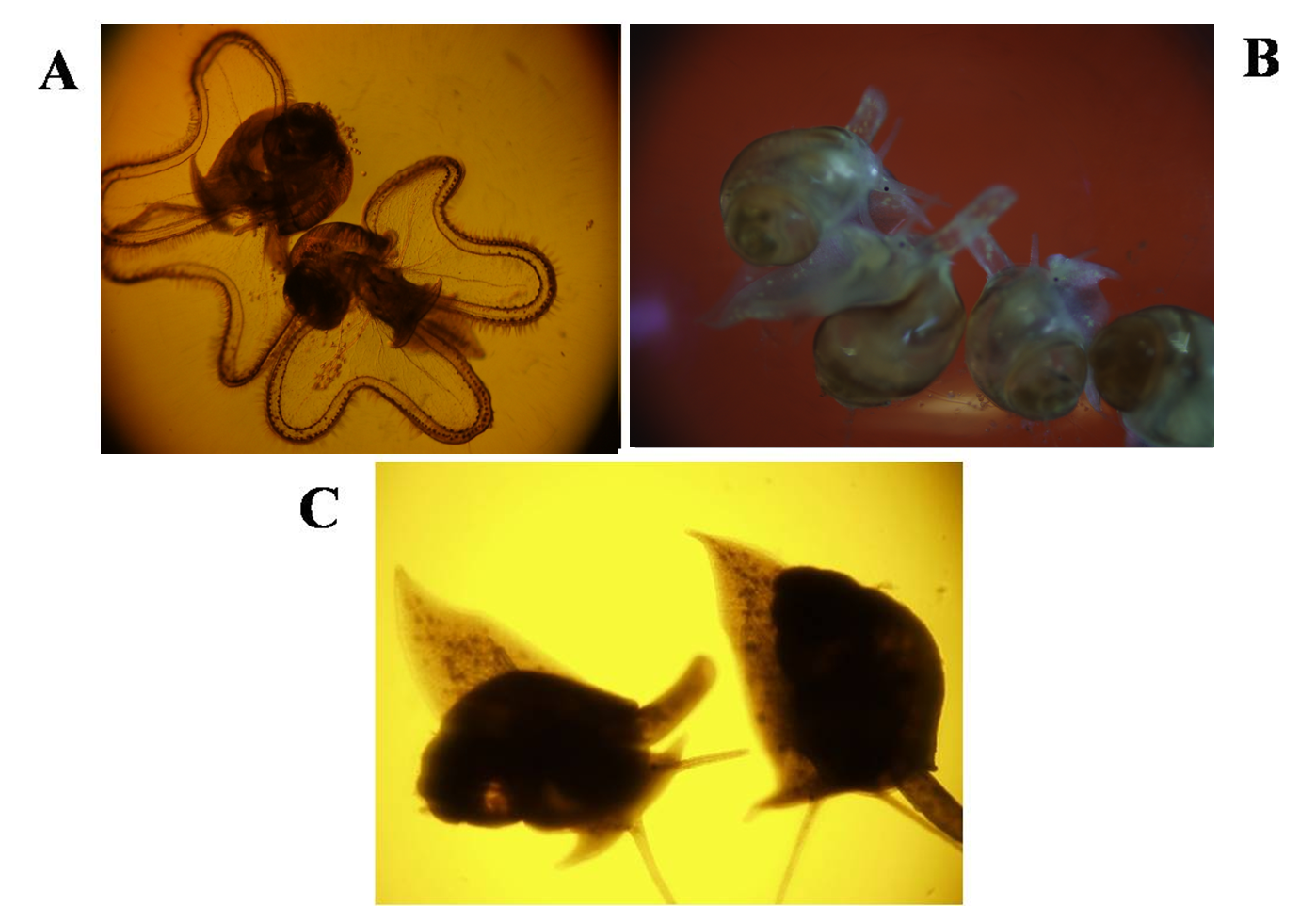

Supplement: Supplementary file 1 — Fig. S1. pCO2 manipulation system (Guo et al., 2015). pCO2 manipulation system with parts as follows 1: air compressor, 2: filter, 3: soda lime column, 4: CaCl2 anhydrate column, 5: disc‐type air filter, 6 and 10: pressure regulation valve, 7 and 11: needle valve, 8 and 12: mass flow sensor, 9: CO2 cylinder, 13: plastic jar, 14: CO2 detector, 15: computer. Solid line: air or CO2 flow; dashed line: digital signal transferring to computer. Fig. S2. Embryonic development of B. areolata. (A) Veliger before attachment; (B) Veliger at the late metamorphosis stage (velum atrophy); (C) Juvenile B. areolata. [file FEB4-9-1503-s001.doc]
