# Supplementary material for: Effects of acidification on the proteome during early development of Babylonia areolata
Source: FEBS Open Bio. 2019 Jul 31;9(9):1503–20. doi: 10.1002/2211-5463.12695 (PMC6722889; doi:10.1002/2211-5463.12695)
Supplement: Supplementary file 2 — Table S1. Differentially expressed proteins with important physiological functions between C1 and E1. [file FEB4-9-1503-s002.doc]

**Supplementary table1** Differentially expressed proteins with important physiological functions between C1and E1

| **Peak Name** | **Protein** | **Species** | **Biological process** | **Cellular component** | **C1 Mean** | **E1 Mean** | **p-value** | **C1/E1 Fold Change** |
| --- | --- | --- | --- | --- | --- | --- | --- | --- |
| gi|51105030 | tumor rejection antigen-like protein | *Lymnaea stagnalis* | protein folding,response to stress | Cytoplasm | 1449245.09 | 83411.34 | 2.77E-07 | 17.37 |
| gi|126697428 | 26S protease regulatory subunit 6B | *Haliotis discus discus* | protein polyubiquitination | Cytoplasm, Nucleus, Proteasome | 46068.53 | 5512.10 | 0.00155 | 8.36 |
| gi|1932827 | pedal peptide precursor protein | *Helix lucorum* | Neuropeptide | neurons | 69380.51 | 17466.20 | 0.00178 | 3.97 |
| cont|000156 | gi|68566311|sp|P0A6N1|EFTU_ECOLI Elongation factor Tu (EF-Tu) (P-43) | *Escherichia coli (contaminant)* | Protein biosynthesis | cytoplasm | 30218.18 | 8444.13 | 0.01373 | 3.58 |
| gi|223868955 | endo-1,3-beta-D-glucanase | *Littorina sitkana* | carbohydrate metabolic process | Cell wall, Secreted | 179947.41 | 56185.43 | 1.93E-06 | 3.20 |
| gi|219806590 | tropomyosin | *Neptunea polycostata* | mitotic cytokinesis | Cytoplasm, Cytoskeleton | 345189.89 | 110938.27 | 0.00108 | 3.11 |
| gi|166406876 | troponin T | *Haliotis diversicolor* | response to calcium ion,muscle filament sliding | striated muscle thin filament,troponin complex | 43239.95 | 14295.85 | 0.30028 | 3.02 |
| gi|126697440 | es1 protein | *Haliotis discus discus* | ATP binding | mitochondrion | 2253.28 | 785.93 | 0.17611 | 2.87 |
| gi|259584272 | ribosomal protein L7A | *Haliotis discus hannai* | translation | ribosome | 3967.83 | 1410.53 | 0.14174 | 2.81 |
| gi|126697348 | Ran-1-prov protein | *Haliotis discus discus* | translation | Nucleus | 278289.68 | 101476.74 | 0.00106 | 2.74 |
| gi|37544573 | myosin heavy chain | *Littorina littorea* | Muscle protein | myosin complex | 1635820.51 | 599381.03 | 5.59E-05 | 2.73 |
| gi|156066422 | calmodulin | *Haliotis diversicolor* | detection of calcium ion,Wnt signaling pathway, calcium modulating pathway | Cytoplasm, Cytoskeleton | 236417.80 | 94751.78 | 0.00065 | 2.50 |
| gi|126697388 | nucleoside diphosphate kinase B | *Haliotis discus discus* | CTP(GTP,UTP) biosynthetic process | Nucleus | 690078.31 | 279713.75 | 2.29E-05 | 2.47 |
| gi|297186112 | poly [ADP-ribose] polymerase 4 | *Aplysia californica* | protein ADP-ribosylation | Nucleus | 53562.69 | 21845.31 | 0.57281 | 2.45 |
| gi|20069098 | 60S ribosomal protein L18 | *Aplysia californica* | translation | ribosome | 232892.74 | 97772.53 | 6.4E-05 | 2.38 |
| gi|260408268 | vitelline envelope zona pellucida domain protein 18 | *Haliotis rufescens* | oocyte development | extracellular space | 1413.78 | 603.18 | 0.05721 | 2.34 |
| gi|61677541 | histone H3 | *Scissurella cf.* | nucleosome assembly | Nucleus | 2468.60 | 1053.44 | 0.01864 | 2.34 |
| gi|60391984 | actin A3 | *Haliotis iris* | Muscle protein | Cytoplasm, Cytoskeleton | 1301837.94 | 579633.04 | 0.10015 | 2.25 |
| gi|20069093 | 40S ribosomal protein S16 | *Aplysia californica* | translation | ribosome | 813630.58 | 364371.51 | 1.99E-05 | 2.23 |
| gi|4519617 | collagen pro alpha-chain | *Haliotis discus* | blood vessel development,embryonic skeletal system development | Extracellular matrix,Secreted | 32919.71 | 65812.07 | 0.02377 | 0.50 |
| gi|157930904 | ubiquitin conjugating enzyme | *Haliotis diversicolor supertexta* | Ubl conjugation pathway | Cytoplasm, Nucleus | 9107.60 | 18245.10 | 0.32759 | 0.50 |
| gi|166406842 | 40S ribosomal protein S3a | *Haliotis diversicolor* | translation | ribosome | 12918.92 | 26051.98 | 0.05558 | 0.50 |
| gi|290751152 | myosin heavy chain type II | *Lepetodrilus pustulosus* | Muscle protein | myosin complex | 5758.89 | 11634.27 | 0.42709 | 0.49 |
| gi|356984565 | peroxiredoxin 6, partial | *Reishia clavigera* | Lipid degradation, Lipid metabolism | Cytoplasm, Cytoplasmic vesicle, Lysosome | 124289.31 | 252158.61 | 0.00278 | 0.49 |
| gi|20069106 | 60S ribosomal protein L31 | *Aplysia californica* | translation | ribosome | 62418.81 | 128093.09 | 0.05627 | 0.49 |
| gi|166406846 | 60S acidic ribosomal protein P0 | *Haliotis diversicolor* | ribosome biogenesis | ribosome | 249547.13 | 513374.05 | 1.76E-05 | 0.49 |
| gi|471279 | kinase-related protein (KRP）-A | *Aplysia californica* | Muscle protein | myosin complex | 33.61 | 70.79 | 0.1582 | 0.47 |
| gi|356983933 | Rab5, partial | *Reishia clavigera* | small GTPase mediated signal transduction | intracellular | 22108.07 | 48060.92 | 0.00376 | 0.46 |
| gi|166406953 | manganese-superoxide dismutase | *Haliotis diversicolor* | response to oxidative stress | Mitochondrion | 33836.87 | 75053.11 | 0.08692 | 0.45 |
| gi|126697334 | calcineurin A | *Haliotis discus discus* | protein dephosphorylation | calcineurin complex | 186051.15 | 425842.94 | 0.00061 | 0.44 |
| gi|290751146 | myosin heavy chain type II | *Cantrainea macleani* | Muscle protein | myosin complex | 17990.28 | 44302.31 | 0.02579 | 0.41 |
| gi|374534595 | mitochondrial ATP synthase beta subunit, partial | *Littorina sp. JV-2012* | ATP synthesis | proton-transporting ATP synthase complex, catalytic core F(1) | 698879.20 | 1741844.88 | 3.98E-05 | 0.40 |
| gi|5588 | type N4 regulatory subunit of protein kinase A | *Aplysia californica* | Cell cycle, Cell division, DNA damage, DNA repair, Meiosis, Mitosis | Cytoplasm, Cytoskeleton, Endosome, Nucleus | 61562.95 | 156862.41 | 0.00043 | 0.39 |
| gi|30515679 | histidine decarboxylase | *Aplysia californica* | cellular amino acid metabolic process | cytosol | 9918.82 | 25372.68 | 0.00078 | 0.39 |
| gi|51038265 | thyroid peroxidase-like protein | *Aplysia californica* | response to oxidative stress | Membrane | 6134.61 | 15806.41 | 0.0007 | 0.39 |
| gi|19852048 | 40S ribosomal protein S29 | *Aplysia californica* | translation | ribosome | 4011.40 | 10339.40 | 0.03064 | 0.39 |
| gi|65307079 | ELAV 2-like protein | *Aplysia californica* | nervous system development,intracellular mRNA localization | Cytoplasm | 184364.19 | 476213.22 | 1.23E-05 | 0.39 |
| gi|154816325 | small G-protein | *Aplysia californica* | small GTPase mediated signal transduction | intracellular,membrane | 7385.41 | 19256.36 | 0.17973 | 0.38 |
| gi|68272051 | p38 MAPK | *Biomphalaria glabrata* | cellular response to cadmium ion,heart morphogenesis,immune response | intracellular | 28502.06 | 82941.46 | 0.07008 | 0.34 |
| gi|13177630 | NCAM-related cell adhesion molecule | *Aplysia californica* | cell adhesion | Membrane | 695.15 | 2099.46 | 0.01429 | 0.33 |
| gi|304441889 | ATP-dependent RNA helicase DDX5 | *Aplysia californica* | Biological rhythms, mRNA processing, mRNA splicing, Transcription, Transcription regulation | Nucleus, Spliceosome | 50253.29 | 156557.31 | 0.00184 | 0.32 |
| gi|829208 | non-neuronal intermediate filament protein A | *Helix aspersa* | single organismal cell-cell adhesion | Cytoplasm, Intermediate filament | 221373.10 | 732339.10 | 7.74E-06 | 0.30 |
| gi|126697438 | ADP-ribosylation factor 2 | *Haliotis discus discus* | small GTPase mediated signal transduction | intracellular | 548.01 | 1874.10 | 0.00121 | 0.29 |
| gi|290751168 | myosin heavy chain type II | *Crepidula fornicata* | Muscle protein | myosin complex | 44161.60 | 153538.95 | 0.03797 | 0.29 |
| gi|166406907 | 60S acidic ribosomal protein P2 | *Haliotis diversicolor* | translational elongation | ribosome | 265673.20 | 1062271.85 | 0.00319 | 0.25 |
| gi|215398875 | LIM protein | *Haliotis discus discus* | heart development,head involution | M band | 6979.25 | 31238.73 | 0.19238 | 0.22 |
| gi|166406844 | ubiquitin-conjugating enzyme | *Haliotis diversicolor* | Ubl conjugation pathway | cytoplasm,nucleus | 632.08 | 2869.57 | 0.17435 | 0.22 |
| gi|211908628 | histone H2A isoform 2 | *Haliotis discus discus* | nucleosome assembly | Nucleus | 42554.42 | 203321.00 | 0.00018 | 0.21 |
| gi|17298377 | cytochrome c oxidase subunit I | *Cochliopina riograndensis* | Transport,Electron transport, Respiratory chain | Membrane, Mitochondrion, Mitochondrion inner membrane | 119.93 | 615.96 | 0.08291 | 0.19 |
| gi|111661543 | catalase | *Haliotis diversicolor supertexta* | response to oxidative stress | Peroxisome | 8622.84 | 48128.71 | 0.01557 | 0.18 |
| gi|126697368 | huntingtin interacting protein K | *Haliotis discus discus* | Apoptosis, Differentiation, Endocytosis, Transcription, Transcription regulation | Cytoplasm, Nucleus | 211.28 | 1188.65 | 0.01511 | 0.18 |
| gi|356984214 | cathepsin D, partial | *Reishia clavigera* | antigen processing and presentation of exogenous peptide antigen via MHC class II | lysosome | 25964.38 | 147986.77 | 8.21E-05 | 0.18 |
| gi|363894934 | putative tubulin beta chain | *Haliotis diversicolor* | microtubule-based process | Cytoplasm, Cytoskeleton | 204638.30 | 1279649.20 | 0.00011 | 0.16 |
| gi|157930910 | glutaredoxin | *Haliotis diversicolor supertexta* | cell redox homeostasis | Mitochondrion | 7879.83 | 55311.38 | 0.06172 | 0.14 |
| gi|158997657 | histone 2A | *Aplysia californica* | nucleosome assembly | Chromosome, Nucleosome core, Nucleus | 289967.41 | 3742722.85 | 1.15E-05 | 0.08 |
| gi|91992378 | vitelline envelope zona pellucida domain 8 | *Haliotis discus hannai* | oocyte development | extracellular space | 4427.35 | 70857.87 | 0.11207 | 0.06 |
