# Supplementary material for: Effects of acidification on the proteome during early development of Babylonia areolata
Source: FEBS Open Bio. 2019 Jul 31;9(9):1503–20. doi: 10.1002/2211-5463.12695 (PMC6722889; doi:10.1002/2211-5463.12695)
Supplement: Supplementary file 3 — Table S2. Differentially expressed proteins with important physiological functions between C2 and E2. [file FEB4-9-1503-s003.doc]

**Supplementary table 2** Differentially expressed proteins with important physiological functions between C2and E2

| **Peak Name** | **Group** | **Species** | **Biological process** | **Cellular component** | **C2 Mean** | **E2 Mean** | **p-value** | **C2/E2 Fold Change** |
| --- | --- | --- | --- | --- | --- | --- | --- | --- |
| gi|71726735 | histone H4 | *Biomphalaria glabrata* | nucleosome assembly | Nucleus | 11122037.85 | 222113.21 | 1.3259E-06 | 50.07 |
| gi|225906407 | heat shock cognate protein 70 | *Haliotis diversicolor* | Stress response | Cell membrane, Cytoplasm, Membrane, Nucleus, Spliceosome | 2628570.97 | 113665.80 | 1.0553E-06 | 23.13 |
| gi|409974552 | H(+)-transporting two-sector ATPase alpha subunit, partial | *Theodoxus transversalis* | ATP synthesis, Hydrogen ion transport | Membrane | 4285271.97 | 222054.50 | 6.3987E-08 | 19.30 |
| gi|268322312 | elongation factor 1 alpha | *Haliotis tuberculata* | Protein biosynthesis | cytoplasm | 904864.29 | 54797.78 | 2.1245E-06 | 16.51 |
| gi|158997661 | histone macro2A.1 | *Aplysia californica* | nucleosome assembly | Nucleus | 87586.55 | 5661.34 | 3.3782E-05 | 15.47 |
| gi|224458718 | CaM kinase II alpha | *Aplysia californica* | response to calcium ion | Cytoplasm, Cytoskeleton | 77736.59 | 6704.54 | 2.7163E-05 | 11.59 |
| gi|301341836 | arginine kinase | *Conus novaehollandiae* | phosphorylation | Cytoplasm | 3042544.89 | 267331.62 | 1.2725E-05 | 11.38 |
| gi|6746611 | malate dehydrogenase precursor | *Nucella lapillus* | Tricarboxylic acid cycle | Mitochondrion | 601978.94 | 56726.66 | 0.00106 | 10.61 |
| gi|51105030 | tumor rejection antigen-like protein | *Lymnaea stagnalis* | protein folding,response to stress | Cytoplasm | 1562340.58 | 148025.34 | 0.13392 | 10.55 |
| gi|20069098 | 60S ribosomal protein L18 | *Aplysia californica* | translation | ribosome | 175132.34 | 18539.50 | 8.6188E-06 | 9.45 |
| gi|91992378 | vitelline envelope zona pellucida domain 8 | *Haliotis discus hannai* | oocyte development | extracellular space | 35238.03 | 4695.09 | 0.39947 | 7.51 |
| gi|20069089 | alpha tubulin 2 | *Aplysia californica* | microtubule-based process | Cytoplasm, Cytoskeleton, Microtubule | 1098281.51 | 155144.88 | 0.00023 | 7.08 |
| gi|166406777 | glutathione-S-transferase isoform | *Haliotis diversicolor* | metabolic process | Cytoplasm, Mitochondrion, Nucleus | 216395.75 | 30601.46 | 4.7151E-06 | 7.07 |
| gi|13647103 | arginine kinase | *Aplysia kurodai* | phosphorylation | Cytoplasm | 207793.10 | 31308.05 | 3.9844E-05 | 6.64 |
| gi|37544573 | myosin heavy chain | *Littorina littorea* | Muscle protein | myosin complex | 1436808.85 | 243562.43 | 0.00058 | 5.90 |
| gi|253771020 | PL10-like protein | *Haliotis asinina* | brain development | Cytoplasm | 1362079.76 | 235459.84 | 0.06877 | 5.78 |
| gi|288938 | RAB2 | *Lymnaea stagnalis* | ER-Golgi transport, Protein transport, Transport | Endoplasmic reticulum, Golgi apparatus, Membrane | 119150.00 | 20633.81 | 2.4476E-05 | 5.77 |
| gi|2706556 | heat shock protein 70 | *Aplysia californica* | Stress response | Cell membrane, Cytoplasm, Membrane, Nucleus, Spliceosome | 886898.47 | 161207.33 | 0.00007515 | 5.50 |
| gi|12620237 | ribosomal protein S6 | *Aplysia californica* | translation | ribosome | 1132029.45 | 213314.71 | 9.6845E-05 | 5.31 |
| gi|126697388 | nucleoside diphosphate kinase B | *Haliotis discus discus* | CTP(GTP,UTP) biosynthetic process | Nucleus | 760237.69 | 164052.34 | 0.00023 | 4.63 |
| gi|71679642 | extracellular regulated kinase | *Littorina littorea* | ATP binding | intracellular | 82571.56 | 18004.84 | 3.1278E-05 | 4.59 |
| gi|126697428 | 26S protease regulatory subunit 6B | *Haliotis discus discus* | protein polyubiquitination | Cytoplasm, Nucleus, Proteasome | 51831.18 | 11585.35 | 0.00897 | 4.47 |
| gi|304367970 | 78 kDa glucose-regulated protein | *Conus novaehollandiae* | cellular response to glucose starvation | Cytoplasm, Endoplasmic reticulum | 438648.91 | 101714.13 | 6.1215E-05 | 4.31 |
| gi|160347070 | ribosomal protein S9 | *Haliotis discus discus* | translation | ribosome | 1091197.00 | 270555.99 | 2.8425E-05 | 4.03 |
| gi|356983730 | galectin, partial | *Reishia clavigera* | synaptic target recognition | cytosol | 238761.16 | 61663.74 | 0.04119 | 3.87 |
| gi|23394914 | alpha-actinin | *Biomphalaria glabrata* | actin filament bundle assembly | Cytoplasm | 735771.23 | 196831.73 | 0.00014 | 3.74 |
| gi|5577 | catalytic subunit of protein kinase A | *Aplysia californica* | cAMP binding | Cytoplasm | 321153.25 | 91175.61 | 0.00077 | 3.52 |
| gi|6746613 | cytosolic malate dehydrogenase precursor | *Nucella lapillus* | tricarboxylic acid cycle | Cytoplasm | 653044.20 | 187347.00 | 0.00024 | 3.49 |
| gi|298108443 | voltage-dependent anion channel 2-like protein | *Haliotis diversicolor* | Ion transport, Transport | mitochondrial outer membrane | 673278.36 | 226775.58 | 0.00294 | 2.97 |
| RRRRRgi|346721863 | REVERSED fibrinogen-related protein 3.2 | *Biomphalaria glabrata* | Adaptive immunity, Blood coagulation, Hemostasis, Immunity, Innate immunity,adipose tissue development,cholesterol metabolic process | Secreted | 25720.33 | 8857.27 | 0.01704 | 2.90 |
| gi|126697436 | transcription factor IIB | *Haliotis discus discus* | Host-virus interaction, Transcription, Transcription regulation | Nucleus | 94816.35 | 35142.11 | 0.00098 | 2.70 |
| cont|000137 | spt|P00760| Cationic trypsin precursor (EC 3.4.21.4) (Beta-trypsin) (Fragment) | *Bos taurus (contaminant)* | Digestion | Secreted | 253750.00 | 98049.33 | 0.01771 | 2.59 |
| gi|4249742 | myosin II heavy chain | *Ilyanassa obsoleta* | Cell cycle, Cell division, Protein transport, Transport | myosin complex | 4095378.34 | 1601143.51 | 5.9059E-05 | 2.56 |
| gi|356983933 | Rab5, partial | *Reishia clavigera* | small GTPase mediated signal transduction | intracellular | 24492.79 | 9739.41 | 0.02361 | 2.51 |
| gi|71370900 | fructose-bisphosphate aldolase, partial | *Crepidula fornicata* | Glycolysis | cytosol | 461213.46 | 183887.91 | 0.00088 | 2.51 |
| gi|297186112 | poly [ADP-ribose] polymerase 4 | *Aplysia californica* | protein ADP-ribosylation | Nucleus | 33286.03 | 14488.11 | 0.58268 | 2.30 |
| gi|126697396 | glutaredoxin 5 | *Haliotis discus discus* | cell redox homeostasis | Mitochondrion | 28850.93 | 12644.50 | 0.05263 | 2.28 |
| gi|126697476 | malate dehydrogenase precursor | *Haliotis discus discus* | Tricarboxylic acid cycle | Mitochondrion | 135651.90 | 61082.64 | 0.01673 | 2.22 |
| gi|379318220 | chaperonin containing T-complex polypeptide subunit zeta | *Haliotis discus hannai* | protein folding | cytoplasm | 175380.16 | 79354.56 | 0.00896 | 2.21 |
| gi|71733130 | ezrin/radixin/moesin | *Aplysia californica* | establishment or maintenance of epithelial cell apical/basal polarity | Cell junction, Cell membrane, Cytoplasm, Cytoskeleton, Membrane | 157061.98 | 72334.34 | 0.00019 | 2.17 |
| gi|154816327 | small G-protein | *Aplysia californica* | small GTPase mediated signal transduction | intracellular,membrane | 182593.22 | 85540.34 | 0.01846 | 2.13 |
| gi|219806590 | tropomyosin | *Neptunea polycostata* | mitotic cytokinesis | Cytoplasm, Cytoskeleton | 844175.52 | 396100.60 | 7.5928E-05 | 2.13 |
| gi|374534595 | mitochondrial ATP synthase beta subunit, partial | *Littorina sp. JV-2012* | ATP synthesis | proton-transporting ATP synthase complex, catalytic core F(1) | 527455.70 | 249343.85 | 0.02235 | 2.12 |
| gi|158997655 | histone 1.1 | *Aplysia californica* | nucleosome assembly | Nucleus | 334734.72 | 159351.86 | 0.55989 | 2.10 |
| gi|126697348 | Ran-1-prov protein | *Haliotis discus discus* | translation | Nucleus | 343107.11 | 166276.06 | 0.00042 | 2.06 |
| gi|418207592 | fructose-biphosphate aldolase, partial | *Haliotis rufescens* | Glycolysis | Glycosome, Peroxisome | 465615.13 | 226661.18 | 0.0183 | 2.05 |
| gi|256550150 | ubiquitin-conjugating enzyme E2N | *Aplysia californica* | Ubl conjugation pathway | cytoplasm,nucleus | 10234.12 | 20426.59 | 0.21833 | 0.50 |
| gi|126697450 | ribosomal protein S14 | *Haliotis discus discus* | translation | ribosome | 835743.53 | 1674676.38 | 1.5247E-05 | 0.50 |
| gi|852074 | myosin regulatory light chain (N-terminus) | *Aplysia californica* | Cell cycle, Cell division | myosin II complex | 15666.06 | 31873.30 | 0.04486 | 0.49 |
| gi|91992392 | vitelline envelope zona pellucida domain 10 | *Haliotis corrugata* | oocyte development | extracellular space | 90744.36 | 187562.77 | 0.22307 | 0.48 |
| gi|13647113 | arginine kinase | *Cellana grata* | phosphorylation | Cytoplasm | 5201.39 | 10816.59 | 0.21767 | 0.48 |
| gi|71370914 | ATP synthase beta subunit, partial | *Haliotis rufescens* | ATP synthesis | proton-transporting ATP synthase complex, catalytic core F(1) | 167341.02 | 359226.17 | 0.03402 | 0.47 |
| gi|126697462 | ribosomal protein S4 | *Haliotis discus discus* | translation | ribosome | 160129.32 | 352397.00 | 0.01718 | 0.45 |
| gi|126697474 | axonemal dynein light chain p33 | *Haliotis discus discus* | Motor protein | Dynein | 99516.46 | 219736.70 | 0.16658 | 0.45 |
| gi|269854565 | Cdc24-like protein | *Biomphalaria glabrata* | small GTPase mediated signal transduction | intracellular | 116039.83 | 266121.52 | 0.00258 | 0.44 |
| gi|126697334 | calcineurin A | *Haliotis discus discus* | protein dephosphorylation | calcineurin complex | 282901.21 | 669695.27 | 0.1575 | 0.42 |
| gi|157072781 | actin depolymerisation factor/cofilin | *Haliotis diversicolor* | actin filament depolymerization | actin cytoskeleton | 68929.95 | 163464.18 | 0.01903 | 0.42 |
| gi|126697460 | ribosomal protein l | *Haliotis discus discus* | translation | ribosome | 193991.91 | 468128.49 | 0.04117 | 0.41 |
| gi|315441240 | vitelline envelope zona pellucida domain 2 type 7 protein | *Haliotis asinina* | oocyte development | extracellular space | 111017.62 | 272194.86 | 0.00669 | 0.41 |
| gi|223868955 | endo-1,3-beta-D-glucanase | *Littorina sitkana* | carbohydrate metabolic process | Cell wall, Secreted | 34042.43 | 84247.69 | 0.06911 | 0.40 |
| gi|89145845 | guanine nucleotide-binding protein G(q), alpha subunit | *Aplysia californica* | adenylate cyclase-modulating G-protein coupled receptor signaling pathway | Membrane | 136659.65 | 340043.01 | 0.00028 | 0.40 |
| gi|166406876 | troponin T | *Haliotis diversicolor* | response to calcium ion,muscle filament sliding | striated muscle thin filament,troponin complex | 52591.49 | 136713.54 | 0.2164 | 0.38 |
| gi|20069093 | 40S ribosomal protein S16 | *Aplysia californica* | translation | ribosome | 578486.51 | 1522612.02 | 0.02636 | 0.38 |
| gi|363894957 | putative polyadenylate-binding protein 1, partial | *Haliotis diversicolor* | mRNA processing, mRNA splicing, Nonsense-mediated mRNA decay | Cytoplasm, Nucleus, Spliceosome | 163374.88 | 435554.73 | 0.15334 | 0.38 |
| gi|34484257 | sodium/potassium ATPase alpha subunit | *Onchidella borealis* | Ion transport, Potassium transport, Sodium transport, Sodium/potassium transport, Transport | Membrane | 127348.07 | 342497.75 | 0.00055 | 0.37 |
| gi|326535851 | protein disulfide isomerase | *Conus imperialis* | cell redox homeostasis | endoplasmic reticulum | 510078.50 | 1441472.44 | 0.00562 | 0.35 |
| gi|34484259 | sodium/potassium ATPase alpha subunit | *Tegula brunnea* | Ion transport, Potassium transport, Sodium transport, Sodium/potassium transport, Transport | Membrane | 50085.08 | 143248.97 | 0.00645 | 0.35 |
| gi|91992368 | vitelline envelope zona pellucida domain 7 | *Haliotis corrugata* | oocyte development | extracellular space | 3376.43 | 9826.19 | 0.1611 | 0.34 |
| gi|126697454 | ribosomal protein l18 | *Haliotis discus discus* | translation | ribosome | 15160.81 | 44310.88 | 0.11246 | 0.34 |
| gi|238481789 | cathepsin L-like cysteine proteinase | *Haliotis diversicolor supertexta* | Digestion | Lysosome | 29691.17 | 86902.39 | 0.04991 | 0.34 |
| gi|322812855 | dynein light chain | *Aplysia californica* | microtubule-based process | dynein complex | 41301.07 | 121523.34 | 0.06329 | 0.34 |
| gi|290751168 | myosin heavy chain type II | *Crepidula fornicata* | Muscle protein | myosin complex | 111434.12 | 341705.22 | 0.00416 | 0.33 |
| gi|126697400 | putative mitochondrial ATP synthase | *Haliotis discus discus* | ATP synthesis | proton-transporting ATP synthase complex, catalytic core F(1) | 95575.62 | 299040.97 | 0.19075 | 0.32 |
| gi|29378341 | munc18-1-interacting protein 1 | *Lymnaea stagnalis* | exocytosis | Cytoplasm | 3898.87 | 12231.06 | 0.11358 | 0.32 |
| gi|126697398 | signal sequence receptor beta-like protein | *Haliotis discus discus* | protein translocation | endoplasmic reticulum | 8599.11 | 27919.43 | 0.37567 | 0.31 |
| gi|158997667 | histone 2B | *Aplysia californica* | nucleosome assembly | nucleus | 1008200.28 | 3331084.94 | 4.1609E-06 | 0.30 |
| gi|9650 | snail soma ferritin | *Lymnaea stagnalis* | Iron storage | Cytoplasm | 22883.95 | 75826.81 | 0.25378 | 0.30 |
| gi|253771018 | vasa-like protein | *Haliotis asinina* | gonad development | cytoplasm,spectrosome | 66465.60 | 223833.89 | 0.21911 | 0.30 |
| gi|126697414 | putative mitochondrial ATP synthase F chain | *Haliotis discus discus* | ATP synthesis | proton-transporting ATP synthase complex, catalytic core F(1) | 23107.96 | 79350.21 | 0.08147 | 0.29 |
| gi|126697420 | protein disulfide isomerase | *Haliotis discus discus* | cell redox homeostasis | endoplasmic reticulum | 70852.29 | 245836.40 | 0.05231 | 0.29 |
| gi|356984214 | cathepsin D, partial | *Reishia clavigera* | antigen processing and presentation of exogenous peptide antigen via MHC class II | lysosome | 35798.69 | 127254.63 | 0.2297 | 0.28 |
| gi|68272051 | p38 MAPK | *Biomphalaria glabrata* | cellular response to cadmium ion,heart morphogenesis,immune response | intracellular | 18049.66 | 66817.19 | 0.00563 | 0.27 |
| gi|166406872 | putative RNA-binding protein | *Haliotis diversicolor* | Transcription, Transcription regulation | Cytoplasm, Nucleus | 164581.66 | 609412.44 | 0.00801 | 0.27 |
| gi|126697402 | histone H3 | *Haliotis discus discus* | rRNA transcription,sexual sporulation resulting in formation of a cellular spore | Nucleus | 2894.65 | 10864.79 | 0.1574 | 0.27 |
| gi|71370922 | triosephosphate isomerase, partial | *Haliotis rufescens* | Gluconeogenesis, Glycolysis, Pentose shunt | extracellular space | 192290.74 | 755031.44 | 0.00837 | 0.25 |
| gi|158997657 | histone 2A | *Aplysia californica* | nucleosome assembly | Chromosome, Nucleosome core, Nucleus | 35543.66 | 140082.19 | 0.00835 | 0.25 |
| gi|215982762 | QM-like protein | *Haliotis diversicolor supertexta* | translation | ribosome | 52191.07 | 205731.37 | 0.07429 | 0.25 |
| gi|312632 | guanine nucleotide regulatory protein beta subunit | *Lymnaea stagnalis* | defense | Membrane | 713151.19 | 3039010.51 | 0.00000844 | 0.23 |
| gi|61677541 | histone H3 | *Scissurella cf. coronata CET-2005* | nucleosome assembly | Nucleus | 1552.62 | 6630.63 | 0.09808 | 0.23 |
| gi|126697354 | thioredoxin peroxidase 1 | *Haliotis discus discus* | cellular response to oxidative stress | Mitochondrion | 4764.11 | 20545.31 | 0.21693 | 0.23 |
| gi|310686606 | ribosomal protein | *Aplysia dactylomela* | translation | ribosome | 69960.11 | 305219.56 | 0.0007 | 0.23 |
| gi|126697456 | ribosomal protein l17 | *Haliotis discus discus* | translation | ribosome | 10281.43 | 45063.82 | 0.10488 | 0.23 |
| gi|304441889 | ATP-dependent RNA helicase DDX5 | *Aplysia californica* | Biological rhythms, mRNA processing, mRNA splicing, Transcription, Transcription regulation | Nucleus, Spliceosome | 147192.76 | 647220.06 | 0.13971 | 0.23 |
| gi|126697448 | proteasome subunit N3 | *Haliotis discus discus* | proteolysis involved in cellular protein catabolic process | Proteasome | 20670.88 | 92889.74 | 0.06873 | 0.22 |
| gi|91992376 | vitelline envelope zona pellucida domain 8 | *Haliotis corrugata* | oocyte development | extracellular space | 12750.22 | 57512.19 | 0.17182 | 0.22 |
| gi|290751152 | myosin heavy chain type II | *Lepetodrilus pustulosus* | Muscle protein | myosin complex | 10006.20 | 45255.99 | 0.18684 | 0.22 |
| gi|431831597 | defender against apopototic cell death 1 | *Haliotis diversicolor* | protein glycosylation | Endoplasmic reticulum,Membrane | 32647.01 | 148354.09 | 0.01135 | 0.22 |
| gi|194473026 | glucose regulated protein 78kDa | *Nacella concinna* | activation of signaling protein activity involved in unfolded protein response,cellular response to glucose starvation | Cytoplasm, Endoplasmic reticulum | 15986.22 | 73496.90 | 3.4575E-05 | 0.22 |
| gi|1932827 | pedal peptide precursor protein | *Helix lucorum* | Neuropeptide | neurons | 61550.66 | 290207.52 | 0.00027 | 0.21 |
| gi|166406842 | 40S ribosomal protein S3a | *Haliotis diversicolor* | translation | ribosome | 13867.62 | 67671.65 | 0.00124 | 0.20 |
| gi|166079862 | synapse-associated protein | *Aplysia californica* | synaptic transmission | synapse | 15814.01 | 79540.47 | 0.00042 | 0.20 |
| gi|51105036 | Bip-like protein | *Lymnaea stagnalis* | lipid metabolic process | Endoplasmic reticulum | 1503.47 | 7597.86 | 0.17871 | 0.20 |
| gi|829208 | non-neuronal intermediate filament protein A | *Helix aspersa* | single organismal cell-cell adhesion | Cytoplasm, Intermediate filament | 52734.02 | 272477.23 | 0.03328 | 0.19 |
| gi|126697482 | alcohol dehydrogenase | *Haliotis discus discus* | ethanol oxidation | Cytoplasm | 62910.00 | 327016.49 | 0.10096 | 0.19 |
| gi|126697446 | RAB protein | *Haliotis discus discus* | small GTPase mediated signal transduction | intracellular | 13056.89 | 68646.88 | 0.02651 | 0.19 |
| gi|12053765 | hemocyanin | *Haliotis tuberculata* | Oxygen transport, Transport | extracellular space | 82839.92 | 440942.17 | 0.03417 | 0.19 |
| gi|156066422 | calmodulin | *Haliotis diversicolor* | detection of calcium ion,Wnt signaling pathway, calcium modulating pathway | Cytoplasm, Cytoskeleton | 704884.28 | 3794574.40 | 8.6114E-05 | 0.19 |
| gi|60391980 | actin A1 | *Haliotis iris* | Muscle protein | Cytoplasm, Cytoskeleton | 585.29 | 3185.46 | 0.06021 | 0.18 |
| gi|16755526 | ribosomal protein L26 | *Littorina littorea* | translation | ribosome | 50867.20 | 276957.21 | 3.8967E-05 | 0.18 |
| gi|156066420 | 60S ribosomal protein L15 | *Haliotis diversicolor* | translation | ribosome | 12122.57 | 67506.52 | 0.17025 | 0.18 |
| gi|27368649 | H2 | *Haliotis tuberculata* | nucleosome assembly | Nucleus | 5166.28 | 28921.97 | 0.27554 | 0.18 |
| gi|126697380 | ATP synthase, H+ transporting, mitochondrial F1 complex, o subunit | *Haliotis discus discus* | ATP synthesis coupled proton transport | membrane | 77876.19 | 476632.87 | 0.33993 | 0.16 |
| gi|73254220 | cytochrome c oxidase subunit 1 | *Reishia clavigera* | Transport | Membrane, Mitochondrion, Mitochondrion inner membrane | 16022.83 | 104867.58 | 0.02083 | 0.15 |
| gi|30313553 | mitochondrial malate dehydrogenase precursor | *Nassarius reticulatus* | Tricarboxylic acid cycle | Mitochondrion | 112525.76 | 773737.32 | 3.2563E-06 | 0.15 |
| gi|71564273 | cadherin like 3 | *Biomphalaria glabrata* | homophilic cell adhesion via plasma membrane adhesion molecules | membrane | 33658.45 | 243201.41 | 0.00012 | 0.14 |
| gi|211908628 | histone H2A isoform 2 | *Haliotis discus discus* | nucleosome assembly | Nucleus | 30137.42 | 220804.88 | 0.11133 | 0.14 |
| gi|56693681 | actin ovestestis isoform | *Aplysia californica* | Muscle protein | Cytoplasm, Cytoskeleton | 176491.59 | 1463617.62 | 0.00049 | 0.12 |
| gi|317120038 | 17 beta-hydroxysteroid dehydrogenase type 11 | *Haliotis diversicolor supertexta* | androgen metabolic process | Endoplasmic reticulum, Membrane, Microsome | 218745.07 | 1829776.00 | 3.1122E-06 | 0.12 |
| gi|126697440 | es1 protein | *Haliotis discus discus* | ATP binding | mitochondrion | 646.05 | 5411.52 | 4.8021E-05 | 0.12 |
| gi|30313537 | mitochondrial malate dehydrogenase precursor | *Littorina littorea* | Tricarboxylic acid cycle | Mitochondrion | 1550.87 | 13187.77 | 0.31084 | 0.12 |
| gi|164604844 | vitellogenin | *Haliotis discus hannai* | Lipid transport, Transport | Secreted,extracellular region | 58151.53 | 502318.15 | 0.1516 | 0.12 |
| gi|157930904 | ubiquitin conjugating enzyme | *Haliotis diversicolor supertexta* | Ubl conjugation pathway | Cytoplasm, Nucleus | 2592.28 | 25078.78 | 0.18505 | 0.10 |
| gi|59895928 | ribosomal protein L28 | *Haliotis asinina* | translation | ribosome | 1524.76 | 16181.71 | 0.00393 | 0.09 |
| gi|363894934 | putative tubulin beta chain | *Haliotis diversicolor* | microtubule-based process | Cytoplasm, Cytoskeleton | 12645.31 | 134285.01 | 2.0412E-05 | 0.09 |
| gi|158635327 | actin | *Haliotis diversicolor* | Muscle protein | Cytoplasm, Cytoskeleton | 79273.29 | 951346.92 | 0.00084 | 0.08 |
| gi|20069087 | alpha tubulin 1 | *Aplysia californica* | microtubule-based process | Cytoplasm, Cytoskeleton, Microtubule | 44399.85 | 578486.48 | 0.00166 | 0.08 |
| RRRRRgi|159239 | REVERSED lysin | *Haliotis cracherodii* | single fertilization,Cell wall biogenesis/degradation | cytoplasm | 339.78 | 5076.68 | 0.00271 | 0.07 |
| gi|126697368 | huntingtin interacting protein K | *Haliotis discus discus* | Apoptosis, Differentiation, Endocytosis, Transcription, Transcription regulation | Cytoplasm, Nucleus | 1224.26 | 21109.10 | 0.08045 | 0.06 |
| gi|166406844 | ubiquitin-conjugating enzyme | *Haliotis diversicolor* | Ubl conjugation pathway | cytoplasm,nucleus | 433.81 | 7967.41 | 0.12656 | 0.05 |
| gi|4519617 | collagen pro alpha-chain | *Haliotis discus* | blood vessel development,embryonic skeletal system development | Extracellular matrix,Secreted | 18271.79 | 338307.03 | 0.01492 | 0.05 |
| gi|13177630 | NCAM-related cell adhesion molecule | *Aplysia californica* | cell adhesion | Membrane | 1250.47 | 25509.43 | 0.04928 | 0.05 |
| gi|51038265 | thyroid peroxidase-like protein | *Aplysia californica* | response to oxidative stress | Membrane | 6657.22 | 157084.53 | 0.22456 | 0.04 |
| gi|893398 | indoleamine dioxygenase-like myoglobin | *Haliotis madaka* | de novo' NAD biosynthetic process from tryptophan | cytoplasm | 1688.48 | 40010.91 | 0.02015 | 0.04 |
| gi|290751146 | myosin heavy chain type II | *Cantrainea macleani* | Muscle protein | myosin complex | 9420.11 | 282710.66 | 7.8807E-05 | 0.03 |
| gi|17298377 | cytochrome c oxidase subunit I | *Cochliopina riograndensis* | Transport,Electron transport, Respiratory chain | Membrane, Mitochondrion, Mitochondrion inner membrane | 1129.30 | 35301.85 | 0.1177 | 0.03 |
| gi|215398875 | LIM protein | *Haliotis discus discus* | heart development,head involution | M band | 4545.49 | 150031.09 | 0.00082 | 0.03 |
| gi|51537343 | cytoplasmic fragile X interacting protein | *Aplysia californica* | Cell shape, Differentiation, Neurogenesis | Cytoplasm | 2347.78 | 78866.63 | 0.04317 | 0.03 |
| gi|443298643 | ferritin | *Concholepas concholepas* | Iron storage | Cytoplasm | 531.00 | 18394.65 | 0.07557 | 0.03 |
| gi|207339266 | Src tyrosine kinase 1 | *Aplysia californica* | Cell adhesion, Cell cycle, Host-virus interaction, Immunity | Cell membrane, Cytoplasm, Cytoskeleton, Membrane, Mitochondrion, Mitochondrion inner membrane, Nucleus | 32549.17 | 1170737.92 | 1.3069E-05 | 0.03 |
| gi|157072783 | elongation factor 1 alpha | *Haliotis diversicolor* | Protein biosynthesis | Cytoplasm, Nucleus | 313.48 | 17208.35 | 0.31007 | 0.02 |
| gi|157930914 | thioredoxin | *Haliotis diversicolor supertexta* | glycerol ether metabolic process | Cytoplasm, Nucleus, Secreted | 1169.49 | 100230.10 | 0.00329 | 0.01 |
| gi|126697438 | ADP-ribosylation factor 2 | *Haliotis discus discus* | small GTPase mediated signal transduction | intracellular | 1382.79 | 148652.15 | 0.10194 | 0.01 |
| gi|30515679 | histidine decarboxylase | *Aplysia californica* | cellular amino acid metabolic process | cytosol | 2731.72 | 303174.78 | 0.32805 | 0.01 |
| gi|471279 | KRP-A | *Aplysia californica* | Muscle protein | myosin complex | 76.94 | 9509.13 | 0.19192 | 0.01 |
| gi|289919162 | ribosomal protein L10a | *Gibbula varia* | translation | ribosome | 923.86 | 142766.57 | 0.03386 | 0.01 |
| gi|332268269 | ATP synthase F0 subunit 6 | *Haliotis iris* | ATP synthesis, Hydrogen ion transport, Ion transport, Transport | Membrane, Mitochondrion | 1929.39 | 312817.76 | 0.02823 | 0.01 |
| gi|5570 | BiP/GRP78 | *Aplysia californica* | protein folding | endoplasmic reticulum | 1175.04 | 357201.89 | 0.01015 | 0.003 |
| gi|125901787 | pol-like protein | *Biomphalaria glabrata* | DNA integration | Membrane | 5937.42 | 6069687.21 | 3.2924E-05 | 0.00098 |
