# Supplementary material for: Effects of acidification on the proteome during early development of Babylonia areolata
Source: FEBS Open Bio. 2019 Jul 31;9(9):1503–20. doi: 10.1002/2211-5463.12695 (PMC6722889; doi:10.1002/2211-5463.12695)
Supplement: Supplementary file 4 — Table S3. Differentially expressed proteins with important physiological functions between C3 and E3. [file FEB4-9-1503-s004.doc]

**Supplementary table 3** Differentially expressed proteins with important physiological functions between C3and E3

| **Peak Name** | **Group** | **Species** | **Biological process** | **Cellular component** | **C3 Mean** | **E3 Mean** | **p-value** | **C3/E3 Fold Change** |
| --- | --- | --- | --- | --- | --- | --- | --- | --- |
| gi|297186112 | poly [ADP-ribose] polymerase 4 | *Aplysia californica* | protein ADP-ribosylation |  | 85679.46 | 2475.90 | 0.00021 | 34.61 |
| gi|194410718 | hemocyanin | *Haliotis diversicolor supertexta* | Oxygen transport, Transport | | 262964.06 | 23187.42 | 0.08611 | 11.34 |
| gi|260408302 | vitelline envelope zona pellucida domain protein 14 | *Haliotis discus hannai* | oocyte development | extracellular space | 257689.16 | 50089.83 | 0.13982 | 5.14 |
| gi|67782245 | importin beta 1 | *Aplysia californica* | intracellular protein transport | intracellular | 176160.21 | 53277.89 | 0.05007 | 3.31 |
| gi|19852048 | 40S ribosomal protein S29 | *Aplysia californica* | translation | ribosome | 28068.02 | 8957.13 | 0.09452 | 3.13 |
| gi|91992376 | vitelline envelope zona pellucida domain 8 | *Haliotis corrugata* | oocyte development | extracellular space | 14886.41 | 4864.03 | 0.36192 | 3.06 |
| gi|27368649 | H2 | *Haliotis tuberculata* | nucleosome assembly | Nucleus | 13437.18 | 4671.93 | 0.33801 | 2.88 |
| gi|51038265 | thyroid peroxidase-like protein | *Aplysia californica* | response to oxidative stress | Membrane | 8355.52 | 2916.28 | 0.23585 | 2.87 |
| gi|260408268 | vitelline envelope zona pellucida domain protein 18 | *Haliotis rufescens* | oocyte development | extracellular space | 1457.94 | 509.09 | 0.14386 | 2.86 |
| gi|16755526 | ribosomal protein L26 | *Littorina littorea* | translation | ribosome | 68375.45 | 25360.80 | 0.01608 | 2.70 |
| gi|126697424 | psmc6 protein | *Haliotis discus discus* | protein catabolic process | cytoplasm | 45117.93 | 17280.86 | 0.01116 | 2.61 |
| gi|30313533 | mitochondrial malate dehydrogenase precursor | *Nucella freycineti* | Tricarboxylic acid cycle | Mitochondrion | 20078.39 | 7807.14 | 0.34195 | 2.57 |
| gi|126697380 | ATP synthase, H+ transporting, mitochondrial F1 complex, o subunit | *Haliotis discus discus* | ATP synthesis coupled proton transport | membrane | 160027.06 | 66606.44 | 0.07695 | 2.40 |
| gi|29378341 | munc18-1-interacting protein 1 | *Lymnaea stagnalis* | exocytosis | Cytoplasm | 5885.57 | 2534.69 | 0.04792 | 2.32 |
| gi|259584272 | ribosomal protein L7A | *Haliotis discus hannai* | translation | ribosome | 3410.53 | 1475.05 | 0.37944 | 2.31 |
| gi|61677541 | histone H3 | *Scissurella cf. coronata CET-2005* | nucleosome assembly | Nucleus | 2785.37 | 1277.44 | 0.00751 | 2.18 |
| gi|356983730 | galectin, partial | *Reishia clavigera* | synaptic target recognition | cytosol | 275512.94 | 129028.13 | 0.38147 | 2.14 |
| gi|166406953 | manganese-superoxide dismutase | *Haliotis diversicolor* | response to oxidative stress | Mitochondrion | 42969.81 | 94632.71 | 0.35479 | 0.45 |
| gi|509413 | twitchin-like protein | *Aplysia californica* | peptidyl-serine phosphorylation | A band | 185715.08 | 419840.68 | 2.08E-05 | 0.44 |
| gi|402170437 | Adh3, partial | *Nucella lapillus* | NADH oxidation | Mitochondrion | 1807.24 | 4465.19 | 0.04969 | 0.40 |
| gi|71370918 | elongation factor 1 alpha, partial | *Haliotis rufescens* | Protein biosynthesis | cytoplasm | 236.23 | 614.37 | 0.12672 | 0.38 |
| gi|326535853 | protein disulfide isomerase | *Conus betulinus* | cell redox homeostasis | endoplasmic reticulum | 26622.68 | 76266.99 | 0.0009 | 0.35 |
| gi|126697446 | RAB protein | *Haliotis discus discus* | small GTPase mediated signal transduction | intracellular | 21097.35 | 63291.53 | 0.44331 | 0.33 |
| gi|256550154 | poly-(ADP-ribose) polymerase I | *Aplysia californica* | protein ADP-ribosylation | nucleus | 3554.01 | 11156.51 | 0.23607 | 0.32 |
| gi|157072783 | elongation factor 1 alpha | *Haliotis diversicolor* | Protein biosynthesis | Cytoplasm, Nucleus | 48.36 | 197.29 | 0.16347 | 0.25 |
| gi|51105030 | tumor rejection antigen-like protein | *Lymnaea stagnalis* | protein folding,response to stress | Cytoplasm | 45963.35 | 1321335.97 | 0.11248 | 0.03 |
