# Supplementary material for: Effects of acidification on the proteome during early development of Babylonia areolata
Source: FEBS Open Bio. 2019 Jul 31;9(9):1503–20. doi: 10.1002/2211-5463.12695 (PMC6722889; doi:10.1002/2211-5463.12695)
Supplement: Supplementary file 5 — Table S4. Differentially expressed proteins with function and process classifications between C2 and E2. [file FEB4-9-1503-s005.doc]

**Supplementary table 4** Differentially expressed proteins with function and process classifications between C2and E2

| **Biological process** | **Up-regulated in E2** | **Down-regulated in E2** |
| --- | --- | --- |
| Translation | 13 | 4 |
| Transcription | 2 | 1 |
| Muscle protein | 7 | 2 |
| Microtubule-based process | 3 | 1 |
| Nucleosome assembly | 5 | 3 |
| Transport | 5 | 3 |
| Transduction | 3 | 2 |
| Oocyte development | 4 | 1 |
| ATP synthesis | 3 | 1 |
| Tricarboxylic acid cycle | 2 | 3 |
| Glycolysis | 1 | 2 |
| Response to calcium ion | 1 | 1 |
| Protein folding | 1 | 1 |
| Protein biosynthesis | 1 | 1 |
| Phosphorylation | 1 | 2 |
| ATP binding | 1 | 1 |
| Digestion | 1 | 1 |
| Ublconjugation pathway | 3 |  |
| Response to oxidative stress | 2 |  |
| Defense | 1 |  |
| Iron storage | 2 |  |
| Cell redox homeostasis | 2 |  |
| Synaptic transmission | 1 |  |
| Cell adhesion | 1 |  |
| Single fertilization,Cell wall biogenesis/degradation | 1 |  |
| Signaling pathway | 1 |  |
| Proteolysis | 1 |  |
| Protein translocation | 1 |  |
| Protein glycosylation | 1 |  |
| Protein dephosphorylation | 1 |  |
| Amino acid metabolic process | 1 |  |
| Lipid metabolism | 1 |  |
| Glycerol ether metabolic process | 1 |  |
| Biosynthetic process | 1 |  |
| Carbohydrate metabolic process | 1 |  |
| ATP synthesis,Transport | 1 |  |
| ATP synthesis coupled proton transport | 1 |  |
| Neuropeptide | 1 |  |
| mRNA processing | 1 |  |
| Motor protein | 1 |  |
| Homophilic cell adhesion via plasma membrane adhesion molecules | 1 |  |
| Heart development,head involution | 1 |  |
| Gonad development | 1 |  |
| Exocytosis | 1 |  |
| Ethanol oxidation | 1 |  |
| Electron transport,Respiratory chain | 1 |  |
| DNA integration | 1 |  |
| Detection of calcium ion,Wnt signaling pathway | 1 |  |
| Cellular response to cadmium ion,heart morphogenesis,immune response | 1 |  |
| Cell shape,Differentiation,Neurogenesis | 1 |  |
| Cell cycle,Cell division | 1 |  |
| Cell adhesion,Cell cycle,Host-virus interaction,Immunity | 1 |  |
| Cell adhesion | 1 |  |
| Blood vessel development,embryonic skeletal system development | 1 |  |
| Biological rhythms,Transcription | 1 |  |
| Apoptosis,Differentiation,Endocytosis,Transcription | 1 |  |
| Antigen processing | 1 |  |
| Androgen metabolic process | 1 |  |
| Activation of signaling protein activity,cellular response to glucose starvation | 1 |  |
| Actin filament depolymerization | 1 |  |
| Actin filament bundle assembly |  | 1 |
| **Stress response** |  | 2 |
| **Immunity, adipose tissue development** |  | 1 |
| **Protein folding,response to stress** |  | 1 |
| Brain development |  | 1 |
| Synaptic target recognition |  | 1 |
| Redox homeostasis |  | 1 |
| Protein polyubiquitination |  | 1 |
| Protein ADP-ribosylation |  | 1 |
| Mitotic cytokinesis |  | 1 |
| Metabolic process |  | 1 |
| Establishment of epithelial cell apical/basal polarity | | 1 |
| Cellular response to glucose starvation |  | 1 |
| CTP(GTP,UTP) biosynthetic process |  | 1 |
| cAMP binding |  | 1 |
