# Supplementary material for: Effects of acidification on the proteome during early development of Babylonia areolata
Source: FEBS Open Bio. 2019 Jul 31;9(9):1503–20. doi: 10.1002/2211-5463.12695 (PMC6722889; doi:10.1002/2211-5463.12695)
Supplement: Supplementary file 6 — Table S5. Differentially expressed proteins enrichment analysis table for profile 3. Table S6. Differentially expressed proteins enrichment analysis table for profile 7. Table S7. Differentially expressed proteins enrichment analysis table for profile 12. Table S8. Differentially expressed proteins enrichment analysis table for profile 13. Table S9. Differentially expressed proteins enrichment analysis table for profile 17. [file FEB4-9-1503-s006.doc]

**Supplementary Table 5** Differentially expressed proteins enrichment analysis table for profile 3

| **Peak Name** | **C1** | **C2** | **C3** | **E1** | **E2** | **E3** | **Proteins name** |
| --- | --- | --- | --- | --- | --- | --- | --- |
| GI363894934 | 0 | -4.02 | -3.62 | 2.64 | -0.61 | -3.53 | putative tubulin beta chain |
| GI71370914 | 0 | -1.31 | -1.48 | 0.04 | -0.21 | -1.77 | ATP synthase beta subunit, partial |
| GI211998646 | 0 | -0.86 | -0.79 | 0.06 | -0.12 | -0.97 | tektin A1 |
| GI126697474 | 0 | -0.81 | -1.44 | -0.03 | 0.33 | -2.2 | axonemal dynein light chain p33 |
| GI4519617 | 0 | -0.85 | -1.28 | 1 | 3.36 | -0.95 | collagen pro alpha-chain |
| GI225580361 | 0 | -0.26 | -0.2 | 0.2 | 0.33 | -0.7 | gelsolin |
| GI30313543 | 0 | -1.23 | -1.61 | -0.14 | -0.84 | -1.19 | mitochondrial malate dehydrogenase precursor |
| GI259685169 | 0 | -0.43 | -0.51 | 0.71 | 0.23 | -0.32 | unnamed protein product |
| GI223868955 | 0 | -2.4 | -2.12 | -1.68 | -1.09 | -2.8 | endo-1,3-beta-D-glucanase |
| GI68272051 | 0 | -0.66 | -0.72 | 1.54 | 1.23 | -0.96 | p38 MAPK |
| GI126697414 | 0 | -1.42 | -0.95 | 0.29 | 0.36 | -0.23 | putative mitochondrial ATP synthase F chain |
| GI829208 | 0 | -2.07 | -1.95 | 1.73 | 0.3 | -1.81 | non-neuronal intermediate filament protein A |
| GI14575745 | 0 | -3.75 | -3.41 | -0.26 | -2.85 | -4.03 | actin |
| GI29378341 | 0 | -2.42 | -1.82 | 0.23 | -0.77 | -3.04 | munc18-1-interacting protein 1 |
| GI256550154 | 0 | -2.51 | -3.17 | 0.9 | -1.93 | -1.52 | poly-(ADP-ribose) polymerase I |

**Supplementary Table 6 Differentially expressed proteins enrichment analysis table for profile 7**

| **Peak Name** | **C1** | **C2** | **C3** | **E1** | **E2** | **E3** | **Proteins name** |
| --- | --- | --- | --- | --- | --- | --- | --- |
| GI164604844 | 0 | -0.78 | -0.54 | -0.2 | 2.33 | 0.36 | vitellogenin |
| CONT000135 | 0 | 0.07 | 0.25 | 0.29 | 4.98 | -0.11 | keratin 1 (epidermolytic hyperkeratosis) |
| GI126697420 | 0 | -0.56 | -0.41 | -0.34 | 1.24 | -0.25 | protein disulfide isomerase |
| GI363894957 | 0 | 0.02 | 0.09 | 0.46 | 1.43 | 0.21 | putative polyadenylate-binding protein 1, partial |
| GI34484259 | 0 | -0.53 | -0.46 | -0.35 | 0.99 | 0.12 | sodium/potassium ATPase alpha subunit |
| GI166406872 | 0 | 0.56 | 0.18 | 0.65 | 2.45 | 0.62 | putative RNA-binding protein |
| GI126697462 | 0 | 0.03 | -0.06 | 0.45 | 1.17 | -0.11 | ribosomal protein S4 |
| GI12053765 | 0 | -0.48 | 0.12 | 0.21 | 1.93 | -0.18 | hemocyanin |
| GI166406842 | 0 | 0.1 | 0.5 | 1.01 | 2.39 | 0.38 | 40S ribosomal protein S3a |
| GI253771018 | 0 | 0.46 | 0.15 | 0.02 | 2.21 | 0.89 | vasa-like protein |
| GI126697488 | 0 | -0.25 | -0.42 | -0.44 | 0.68 | -0.12 | ribosomal protein l5 |
| GI126697448 | 0 | -0.19 | 0.2 | -0.22 | 1.98 | 0.42 | proteasome subunit N3 |
| GI326535851 | 0 | -0.93 | -0.86 | -0.6 | 0.57 | 0.06 | protein disulfide isomerase |
| GI20069087 | 0 | -1.06 | -1.18 | 0.36 | 2.65 | -0.73 | alpha tubulin 1 |
| GI238481789 | 0 | -0.59 | -0.23 | 0.1 | 0.96 | 0.25 | cathepsin L-like cysteine proteinase |
| GI211908628 | 0 | -0.5 | 0.72 | 2.26 | 2.38 | 0.36 | histone H2A isoform 2 |
| GI5570 | 0 | -1.14 | -0.36 | -0.75 | 7.11 | -1.29 | BiP/GRP78 |
| GI156066420 | 0 | -0.44 | -0.06 | 0.04 | 2.04 | 0.7 | 60S ribosomal protein L15 |
| GI126697456 | 0 | -0.07 | -0.15 | 0.39 | 2.06 | 0.24 | ribosomal protein l17 |
| GI157930914 | 0 | 0.09 | 0.69 | 0.35 | 6.51 | -0.2 | thioredoxin |
| GI51537343 | 0 | -0.14 | -0.01 | -0.78 | 4.94 | -0.85 | cytoplasmic fragile X interacting protein |
| GI59895928 | 0 | 0.04 | 0.65 | -0.58 | 3.44 | -0.2 | ribosomal protein L28 |
| GI126697482 | 0 | 0.22 | 0.05 | -0.31 | 2.6 | 0.18 | alcohol dehydrogenase |
| GI332268269 | 0 | 1.38 | 1.76 | 0.17 | 8.72 | 1.59 | ATP synthase F0 subunit 6 |
| GI431831597 | 0 | -0.21 | 0.46 | -0.81 | 1.98 | 0.16 | defender against apopototic cell death 1 |
| GI194473026 | 0 | -1.22 | 0.11 | -0.5 | 0.98 | -0.6 | glucose regulated protein 78kDa |
| GI126697438 | 0 | 1.34 | 0.91 | 1.77 | 8.08 | 1.26 | ADP-ribosylation factor 2 |
| GI317120038 | 0 | 0.09 | -0.19 | -0.23 | 3.16 | -0.34 | 17 beta-hydroxysteroid dehydrogenase type 11 |
| GI126697354 | 0 | 0.41 | 0.57 | 0.15 | 2.52 | 0.65 | thioredoxin peroxidase 1 |
| GI157930904 | 0 | -1.81 | 0.41 | 1 | 1.46 | 0.43 | ubiquitin conjugating enzyme |
| GI158635327 | 0 | -1.29 | -1.07 | -0.95 | 2.29 | -1.45 | actin |
| GI471279 | 0 | 1.19 | 1.52 | 1.07 | 8.14 | 2.15 | KRP-A |
| GI125901787 | 0 | -0.64 | 1.24 | -0.51 | 9.36 | 1.58 | pol-like protein |
| GI126697440 | 0 | -1.8 | -0.72 | -1.52 | 1.26 | -1.03 | es1 protein |
| GI443298643 | 0 | -0.07 | -0.09 | -0.99 | 5.05 | 0.1 | ferritin |
| GI13177630 | 0 | 0.85 | 1.14 | 1.59 | 5.2 | 0.63 | NCAM-related cell adhesion molecule |
| GI30515679 | 0 | -1.86 | -1.44 | 1.36 | 4.93 | -0.48 | histidine decarboxylase |
| GI207339266 | 0 | -1 | -1.22 | 0.16 | 4.17 | -0.43 | Src tyrosine kinase 1 |
| GI290751146 | 0 | -0.93 | -0.23 | 1.3 | 3.97 | 0.42 | myosin heavy chain type II |
| GI30313537 | 0 | -0.11 | 0.23 | -0.08 | 2.98 | 0.29 | mitochondrial malate dehydrogenase precursor |
| GI166406844 | 0 | -0.54 | -0.18 | 2.18 | 3.66 | -0.31 | ubiquitin-conjugating enzyme |
| GI51038265 | 0 | 0.12 | 0.45 | 1.37 | 4.68 | -1.07 | thyroid peroxidase-like protein |
| GI215398875 | 0 | -0.62 | 1.26 | 2.16 | 4.43 | 1.19 | LIM protein |
| GI71564273 | 0 | 0.03 | -0.25 | -0.08 | 2.88 | -0.73 | cadherin like 3 |
| GI310686606 | 0 | -1.55 | -1.16 | -0.11 | 0.58 | -0.27 | ribosomal protein |
| GI893398 | 0 | -0.61 | 0.1 | 0.15 | 3.96 | -0.14 | indoleamine dioxygenase-like myoglobin |

**Supplementary Table 7** Differentially expressed proteins enrichment analysis table for profile 12

| **Peak Name** | **C1** | **C2** | **C3** | **E1** | **E2** | **E3** | **Proteins name** |
| --- | --- | --- | --- | --- | --- | --- | --- |
| GI225906407 | 0 | -0.54 | -0.54 | -0.41 | -5.08 | -0.86 | heat shock cognate protein 70 |
| GI253771020 | 0 | -0.63 | -0.68 | -0.5 | -3.16 | -1.32 | PL10-like protein |
| GI379318220 | 0 | 0.27 | -0.06 | 0.01 | -0.88 | 0.28 | chaperonin containing T-complex polypeptide subunit zeta |
| GI298108443 | 0 | 0.05 | 0 | -0.44 | -1.52 | 0.01 | voltage-dependent anion channel 2-like protein |
| GI71726735 | 0 | 1.94 | 2.07 | 0.58 | -3.71 | 2.03 | histone H4 |
| GI4249742 | 0 | 0.12 | 0.12 | 0.35 | -1.24 | 0.51 | myosin II heavy chain |
| GI301341836 | 0 | -0.48 | -0.33 | -0.36 | -3.99 | -0.38 | arginine kinase |
| GI160347070 | 0 | -0.42 | -0.72 | -0.49 | -2.43 | -0.89 | ribosomal protein S9 |
| GI126697388 | 0 | 0.14 | -0.19 | -1.3 | -2.07 | -0.1 | nucleoside diphosphate kinase B |
| GI363894901 | 0 | 0.8 | -0.17 | -0.64 | -0.15 | -0.13 | putative 60S ribosomal protein L3 |
| GI409974552 | 0 | -0.32 | -0.47 | -0.32 | -4.59 | -0.53 | H(+)-transporting two-sector ATPase alpha subunit, partial |
| GI6746611 | 0 | -0.62 | -0.78 | -0.12 | -4.03 | -1.3 | malate dehydrogenase precursor |
| GI356983730 | 0 | 0.1 | 0.3 | 0.14 | -1.86 | -0.79 | galectin, partial |
| GI158997655 | 0 | 0.21 | 0.24 | -0.71 | -0.86 | -0.23 | histone 1.1 |
| GI6746613 | 0 | 0.54 | 0.49 | 0.28 | -1.26 | 0.63 | cytosolic malate dehydrogenase precursor |
| GI37544573 | 0 | -0.19 | -0.24 | -1.45 | -2.75 | -0.22 | myosin heavy chain |
| GI12620237 | 0 | -0.34 | -0.74 | -0.36 | -2.74 | -0.66 | ribosomal protein S6 |
| GI288938 | 0 | 0.44 | 0.88 | 0.44 | -2.09 | 0.49 | RAB2 |
| GI224458718 | 0 | -0.13 | -0.43 | -0.92 | -3.67 | -0.45 | CaM kinase II alpha |
| GI5577 | 0 | 0.41 | 0.63 | 0.06 | -1.4 | 0.65 | catalytic subunit of protein kinase A |
| GI20069098 | 0 | -0.41 | -0.78 | -1.25 | -3.65 | -0.99 | 60S ribosomal protein L18 |
| GI268322312 | 0 | -0.27 | -0.35 | -0.22 | -4.32 | -0.23 | elongation factor 1 alpha |
| GI158997661 | 0 | 0.09 | 0.35 | 0.74 | -3.86 | 0.37 | histone macro2A.1 |

**Supplementary Table 8** Differentially expressed proteins enrichment analysis table for profile 13

| **Peak Name** | **C1** | **C2** | **C3** | **E1** | **E2** | **E3** | **Proteins name** |
| --- | --- | --- | --- | --- | --- | --- | --- |
| GI71370918 | 0 | 1.16 | -1.11 | 0.52 | 1.55 | 0.27 | elongation factor 1 alpha, partial |
| GI9954249 | 0 | 0.54 | 0.17 | -0.15 | 1.51 | 0.39 | tropomyosin |
| GI30313533 | 0 | 1.63 | 0.91 | 0.09 | 1.97 | -0.45 | mitochondrial malate dehydrogenase precursor |
| GI215982762 | 0 | 0.72 | 0.58 | 0.47 | 2.7 | 0.4 | QM-like protein |
| GI126697450 | 0 | -0.27 | -0.43 | -0.57 | 0.73 | -0.76 | ribosomal protein S14 |
| GI156066422 | 0 | 1.58 | 1.21 | -1.32 | 4 | 0.5 | calmodulin |
| GI126697366 | 0 | 0.54 | 0.1 | 0.49 | 1.38 | 0.66 | l-3-hydroxyacyl-coenzyme a dehydrogenase, short chain |
| GI157072783 | 0 | 0.85 | -1.84 | 0.2 | 6.63 | 0.19 | elongation factor 1 alpha |
| GI56693681 | 0 | 0.55 | 0.67 | -0.51 | 3.6 | 0.11 | actin ovestestis isoform |
| GI146428671 | 0 | 0.64 | 0.23 | 0.12 | 1.13 | 0.76 | Cu/Zn-superoxide dismutase Haliotis diversicolor supertexta |
| GI91992392 | 0 | 0.38 | -0.24 | -0.55 | 1.42 | -0.28 | vitelline envelope zona pellucida domain 10 |
| GI20069106 | 0 | 2.11 | 1.15 | 1.04 | 2.06 | 1.93 | 60S ribosomal protein L31 |
| GI408778253 | 0 | 0.19 | 0.12 | -0.5 | 0.56 | -0.38 | prohibitin-2, partial |
| GI269854565 | 0 | 0.45 | -0.03 | -0.09 | 1.65 | 0.18 | Cdc24-like protein |
| GI53801569 | 0 | 0.1 | -0.31 | -0.44 | 0.44 | -0.79 | NaK-ATPase alpha subunit, partial |
| GI322812855 | 0 | 0.99 | 0.49 | 0.16 | 2.54 | 0.78 | dynein light chain |
| GI30088884 | 0 | 0.75 | 0.07 | 0.05 | 1.2 | -0.3 | beta tubulin |
| GI34484257 | 0 | 0.02 | 0.11 | -0.59 | 1.45 | -0.15 | sodium/potassium ATPase alpha subunit |
| GI290751130 | 0 | 0.93 | 0.67 | 0.09 | 1.36 | 0.95 | myosin heavy chain type II |
| GI73254220 | 0 | 1.02 | 1.01 | -0.24 | 3.73 | 0.83 | cytochrome c oxidase subunit 1 |
| GI166406876 | 0 | 0.28 | -0.23 | -1.6 | 1.66 | -0.27 | troponin T |
| GI89145845 | 0 | 0.55 | 0.45 | 0.1 | 1.87 | 0.44 | guanine nucleotide-binding protein G(q), alpha subunit |
| GI20069093 | 0 | -0.49 | -0.94 | -1.16 | 0.9 | -0.83 | 40S ribosomal protein S16 |
| GI289919162 | 0 | 2.05 | 1.54 | 0.68 | 9.33 | 2.47 | ribosomal protein L10a |
| GI25140448 | 0 | 1.27 | -0.34 | 0.89 | 1.59 | -0.1 | 40S ribosomal protein-like protein |
| GI126697398 | 0 | 0.43 | -0.48 | -0.74 | 2.13 | 0.47 | signal sequence receptor beta-like protein |
| GI194350623 | 0 | 0.74 | 0.67 | -0.26 | 1.46 | 0.04 | glutathione S-transferase |
| RRRRRGI159239 | 0 | 1.95 | 1.47 | 0.8 | 5.85 | 1.37 | - |
| GI91992368 | 0 | 2.63 | 1.94 | 0.61 | 4.17 | 1.35 | vitelline envelope zona pellucida domain 7 |
| GI126697378 | 0 | 0.68 | 0.19 | -0.08 | 1.18 | -0.24 | mitochondrial ATP synthase delta chain |
| GI51105036 | 0 | -0.31 | -1.01 | -0.81 | 2.03 | -1.51 | Bip-like protein |
| GI17298377 | 0 | 3.24 | 1.86 | 2.36 | 8.2 | 1.01 | cytochrome c oxidase subunit I |
| GI30313553 | 0 | 0.55 | 0.4 | 0.21 | 3.33 | -0.3 | mitochondrial malate dehydrogenase precursor |
| GI1932827 | 0 | -0.17 | -0.53 | -1.99 | 2.06 | -0.78 | pedal peptide precursor protein |

**Supplementary Table 9** Differentially expressed proteins enrichment analysis table for profile 17

| **Peak Name** | **C1** | **C2** | **C3** | **E1** | **E2** | **E3** | **Proteins name** |
| --- | --- | --- | --- | --- | --- | --- | --- |
| GI60391980 | 0 | 0.87 | 1.41 | -0.02 | 3.31 | 0.49 | actin A1 |
| CONT000125 | 0 | 0.66 | 0.88 | -0.61 | 2.6 | 0.76 | Keratin, type II cytoskeletal 6A (Cytokeratin 6A) (CK 6A) (K6a keratin) |
| GI126697402 | 0 | 1.31 | 0.95 | 0.98 | 3.22 | 1.34 | histone H3 |
| GI126697380 | 0 | 0.15 | 1.19 | 0.01 | 2.77 | -0.07 | ATP synthase, H+ transporting, mitochondrial F1 complex, o subunit |
| GI315441240 | 0 | 0.75 | 1.6 | 0.87 | 2.04 | 1.05 | vitelline envelope zona pellucida domain 2 type 7 protein |
| GI71370922 | 0 | 0.76 | 1.01 | 0.76 | 2.73 | 0.44 | triosephosphate isomerase, partial |
| GI312632 | 0 | 0.56 | 0.62 | 0.33 | 2.65 | 0.52 | guanine nucleotide regulatory protein beta subunit |
| GI126697424 | 0 | 1.38 | 2.39 | 0.4 | 1.27 | 1 | psmc6 protein |
| GI126697362 | 0 | 0.45 | 1.08 | -0.17 | 1.12 | 0.76 | myosin essential light chain |
| GI16755526 | 0 | 0.89 | 1.32 | -0.22 | 3.34 | -0.11 | ribosomal protein L26 |
| GI91992376 | 0 | 1.07 | 1.29 | 0.23 | 3.24 | -0.32 | vitelline envelope zona pellucida domain 8 |
| GI126697400 | 0 | 0.58 | 0.8 | 0.64 | 2.22 | 0.45 | putative mitochondrial ATP synthase |
| GI260408274 | 0 | 0.37 | 0.4 | 0.38 | 1.22 | 0.61 | vitelline envelope zona pellucida domain protein 21 |
| GI126697334 | 0 | 0.6 | 0.69 | 1.19 | 1.85 | 0.7 | calcineurin A |
| GI290751168 | 0 | 1.34 | 2.21 | 1.8 | 2.95 | 2.25 | myosin heavy chain type II |
| GI9650 | 0 | 0.42 | 0.8 | 0.25 | 2.15 | 0.95 | snail soma ferritin |
| GI158997667 | 0 | 2.3 | 2.65 | 0.97 | 4.02 | 2.32 | histone 2B |
| GI304441889 | 0 | 1.55 | 1.46 | 1.64 | 3.69 | 1.16 | ATP-dependent RNA helicase DDX5 |
| GI126697368 | 0 | 2.53 | 2.34 | 2.49 | 6.64 | 2.23 | huntingtin interacting protein K |
| GI126697390 | 0 | 1.42 | 1.5 | 0.55 | 1.94 | 1.99 | ubiquitin-like protein |
| GI290751152 | 0 | 0.8 | 0.83 | 1.01 | 2.97 | 1.23 | myosin heavy chain type II |
| GI410519429 | 0 | 0.55 | 1.15 | -0.52 | 1.26 | 0.47 | cathepsin L |
| GI157930920 | 0 | 0.85 | 1.76 | 0.87 | 1.43 | 1.09 | splicing factor arginine/serine-rich 4 |
| GI27368649 | 0 | 1.18 | 2.56 | 0.12 | 3.66 | 1.03 | H2 |
| GI19852048 | 0 | 2.42 | 2.81 | 1.37 | 2.8 | 1.16 | 40S ribosomal protein S29 |
